# Impairment of neuronal activity occurs at the early stages of the aggregation cascade of Aβ_1-42_ and mutant Tau

**DOI:** 10.1242/dmm.052295

**Published:** 2026-04-01

**Authors:** Franziska Hirsch, Nino F. Läubli, Anushree Kelkar, Mira Sleiman, Yvonne Woitzat, Christian Gallrein, Vanessa Gumz, Gurleen Kaur Kalsi, Ana Fernandez-Villegas, Gabriele S. Kaminski Schierle, Janine Kirstein

**Affiliations:** ^1^ Department of Cell Biology, University of Bremen, Leobener Strasse 2, 28359 Bremen, Germany; ^2^ Molecular Neuroscience Group, Department of Chemical Engineering and Biotechnology, University of Cambridge, Philippa-Fawcett Drive, Cambridge CB3 0AS, UK; ^3^ Leibniz Institute on Aging – Fritz-Lipmann-Institute, Beutenbergstrasse 11, 07745 Jena, Germany; ^4^ Friedrich-Schiller-Universität, Institute for Biochemistry & Biophysics, Hans Knöll-Strasse 2, 07745 Jena, Germany

**Keywords:** *C. elegans*, Alzheimers disease, Tau, Abeta, Neurodegeneration, Aging

## Abstract

Alzheimer's disease (AD) is a progressive neurodegenerative disease that is characterized by the accumulation of amyloid-β (Aβ) plaques and neurofibrillary Tau tangles, ultimately leading to brain atrophy and death. To elucidate the relationship between the aberrant folding and aggregation of Aβ and mutant Tau and neuronal function, we monitored neuronal activity in *C. elegans* AD models across age. For that, we used a neuronal GCaMP reporter to monitor fluctuations in Ca^2+^ and developed microfluidic devices to immobilize nematodes to non-invasively assess neuronal activity. Our findings revealed that expression of both Aβ and Tau lead to significant reductions in neuronal activity and function in young adult animals, preceding the accumulation of amyloid aggregates. Notably, Aβ expression and aggregation in muscle tissue produced detrimental effects on neuronal activity comparable to those seen after expression in neurons, suggesting that proteotoxic stress in muscle can influence neuronal function. This may occur through propagation of Aβ from muscle to neurons or through retrograde signaling pathways. Further, our new sub-stoichiometrically labeled Tau strains highlight the significant impact Tau^P301L,V337M^ has on neuronal activity throughout aging. These results enhance our understanding of the early functional effects of amyloid aggregation in Alzheimer's disease.

## Teaser

**Summary:** Loss of neuronal activity occurs early before amyloid fibrils accumulate in neurodegenerative disease *C. elegans* models that pan-neuronally express Alzheimer pathology-associated human amyloid-β and Tau.

## INTRODUCTION

Alzheimer's disease (AD) is the most common form of dementia and characterized by a progressive decrease in neuronal activity and severe neurodegeneration. The neuropathological hallmarks of AD are the accumulation of amyloid-β (Aβ) plaques and formation of neurofibrillary Tau tangles. However, in the early presymptomatic stages of the disease, neuronal hyperactivity of cortical and hippocampal brain areas has been observed in sporadic and familial forms of AD (Bookheimer et al., 2000; Dickerson et al., 2005). Several factors can lead to the hyperexcitability of selected neurons and neuronal circuits in AD, including perturbed Ca^2+^ homeostasis, NMDA receptor activation, Aβ and Tau themselves, as well as genetic risk factors, such as apolipoprotein E (APOE) and presenilin 1 and 2 (PSEN1 and PSEN2, respectively) (Targa Dias Anastacio et al., 2022). Thus far, it is not understood how hyperexcitability eventually leads to reduced neuronal activity. As neuronal activity promotes the production and secretion of Aβ, by e.g. activating endocytosis (Cataldo et al., 2000), the hyperexcitability in the early stages of AD could be a pathogenic driver of the disease. However, in contrast to murine Aβ models, Tau^P301L^-expressing mice do not show a hyperexcitability and are marked by a loss of excitatory neuron function (Fu et al., 2017; Martinsson et al., 2022). Additionally, transgenic mouse models that express mutant Tau^P301S^ show impaired synaptic function in the hippocampus, and mutant Tau^P301S,G272V^ mice display axonopathy without overt neuronal loss before fibrillary Tau tangles are formed (Leroy et al., 2007; Yoshiyama et al., 2007). Nevertheless, despite an increase in the number of studies on AD pathology in different model systems, it is still poorly understood how expression and aberrant folding, and amyloid fibril formation of Aβ and mutant Tau leads to neurodegeneration.

In this study, we aimed to elucidate how expression and aggregation of human Aβ and Tau in *C. elegans* are linked to neuronal activity as aging and AD pathology progress. For that, we used *C. elegans* Aβ and Tau models, analyzed neuronal activity, function, and organismal physiology over the course of Aβ and Tau aggregation. Neuronal activity was measured using a genetically encoded Ca^2+^ sensor, GCaMP6m. To facilitate a rapid and reliable assessment of neuronal activity of our AD *C. elegans* models, we established a microfluidic device that permits the trapping and immobilization of living nematodes in a non-invasive manner.

We have further established a new sub-stoichiometrically fluorescently tagged neuronal Tau pathology model in *C. elegans* that carries two patient-derived point mutations, P301L and V337M (Barghorn et al., 2004; Crowther et al., 1989). The pan-neuronal expression of Tau^P301L,V337M^ led to age-dependent aggregation, cell-cell propagation, and severe proteotoxicity on cellular and organismal level. Using our previously established Aβ (Gallrein et al., 2021) and the novel Tau model, we found that both AD-associated amyloid proteins lead to a significant decrease of neuronal activity and function already in young adults, i.e. before an accumulation of amyloid aggregates. Importantly, neuronal activity is equally impaired when the amyloid protein is expressed in a peripheral tissue, suggesting a retrograde signaling of the proteotoxic challenge to the neurons.

## RESULTS

### Microfluidic devices permit the study of neuronal activity in living animals by quantifying GCaMP6m fluorescence intensity levels

In our study, we aimed to assess neuronal activity in Alzheimer's disease models that express the Aβ_1-42_ peptide as well as wild-type Tau (Tau^WT^) and mutant Tau (Tau^P301L,V337M^), to correlate the aggregation propensity and proteotoxicity of Aβ_1-42_ and Tau with their effect on neuronal activity and function.

**Fig. 1.**
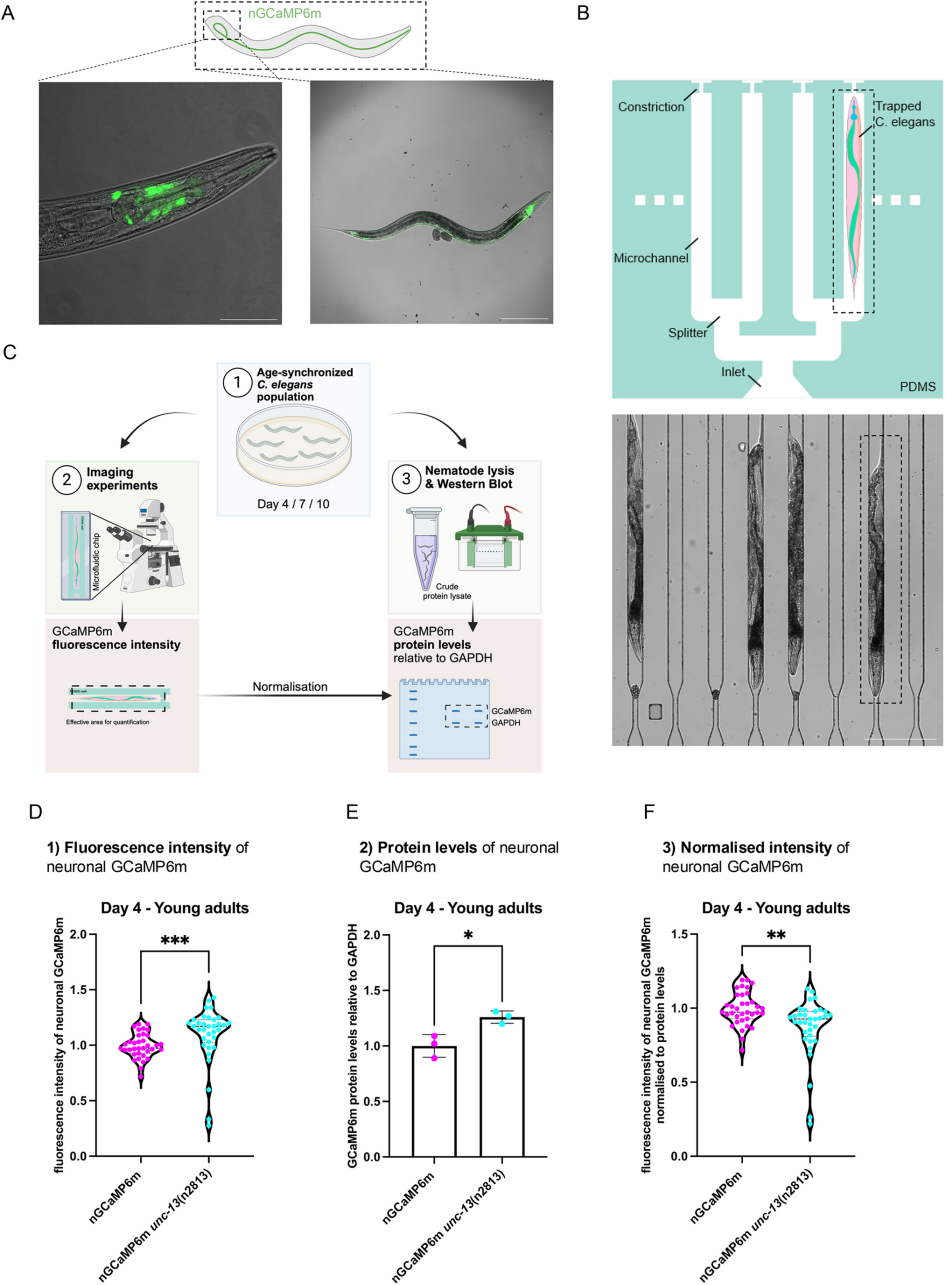
**Proof of concept – assessment of neuronal activity by quantifying GCaMP6m intensity levels of living animals in microfluidic devices.** (A) Representative confocal fluorescent images of a young adult animal (4 days old) of the novel pan-neuronal GCaMP6m strain (nGCaMP6m). The image on the right is 100-fold magnified. Scale bar: 200 μm. The image on the left shows the respective close-up of the head region 400-fold magnified. Scale bar: 50 μm. (B) Schematic representation of the design of the polydimethylsiloxane (PDMS) microfluidic device used for the imaging of living *C. elegans* (top image). Animals enter the device at the inlet by gravity and are sucked into the channels by applying gentle negative pressure. The constriction prevents animals from escaping at the opposing side. Inside the channels the animals are mechanically trapped along their body and *C. elegans* are straightened. The device allows imaging of multiple animals simultaneously. The image depicted at the bottom is 100-fold magnified. Scale bar: 200 µm. (C) Workflow for the quantification of GCaMP6m fluorescence intensity in living *C. elegans* as a readout for the assessment of neuronal activity. Generation of age-synchronized cohorts of young adults (day 4 of life) and older animals (day 7 and day 10 of life) (1). Imaging experiments are performed using PDMS microfluidic devices to record GCaMP6m fluorescence intensities from alive animals. In the subsequent analysis step GCaMP6m fluorescence intensities are quantified from single snapshot images. Age-synchronized nematodes are lysed to obtain the crude protein fraction (2). Lysates are analyzed by western blots. Neuronal GCaMP6m protein levels are normalized to GAPDH protein levels of the whole animal that are then used to normalize GCaMP6m fluorescence intensities. (D) Violine dot plot of the average GCaMP6m fluorescence of young adult animals (4 days old) of the neuronal GCaMP6m strain (nGCaMP6m) and the nGCaMP6m *unc-13*(n2813) mutant strain. The mutant *unc*-13(n2813) (strain MT8004) was crossed with the nGCaMP6 strain and GCaMP6m intensities were measured in animals using microfluidic devices with a widefield fluorescence microscope as outlined in A and C. GCaMP6m intensities were quantified using Fiji. Every dot represents the neuronal GCaMP6m fluorescence intensity of a single animal of nGCaMP6m (magenta) and nGCaMP6m *unc-13*(n2813) (turquoise). *n*=3 and *N*=35 animals (with *n*=number of performed replicates and *N*=total number of analyzed animals combined from all replicates). Mann–Whitney test was performed to assess significance (***P≤0.001). (E) Quantification of GCaMP6m proteins levels by western blot from crude protein lysates of young adult (4 days old) nGCaMP6m (magenta) and nGCaMP6m *unc-13*(n2813) (turquoise) animals. Scatter dot plot shows quantification of GCaMP6m protein levels relative to whole nematode GAPDH from three independent cohorts. Unpaired Student's *t*-test with Welch's correction was performed to assess significance (*P≤0.05). (F) Violine dot plot of the average GCaMP6m fluorescence intensity normalized to the GCaMP6m protein level of young adult animals (4 days old) of the neuronal GCaMP6m strain (nGCaMP6m) and the nGCaMP6m *unc-13*(n2813) mutant strain. GCaMP6m intensities as depicted in (D) were multiplied with the ratio between nGCaMP6m protein levels of the nGCaMP6m strain and nGCaMP6m *unc-13*(n2813) mutant strain. Every dot represents the neuronal GCaMP6mGCaMP6m fluorescence intensity normalized to GCaMP6m protein levels of a single animal of nGCaMP6m (magenta) and nGCaMP *unc-13*(n2813) (turquoise). *n*=3 and *N*=35 animals. Mann–Whitney test was performed to assess significance (**P≤0.01).

Neuronal activity can be analyzed using Ca^2+^-dependent fluorescence measurements by applying genetically encoded Ca^2+^ indicators, such as GCaMP (Tian et al., 2012). Neuronal action potentials lead to an influx of Ca^2+^ ions through voltage-gated Ca^2+^ channels, this results in a rapid increase in intracellular Ca^2+^ concentrations that are sensed by the GCaMP sensor and can be used as a proxy for neuronal activity (Chen et al., 2013). We have generated a *C. elegans* neuronal GCaMP strain that expresses GCaMP6m pan-neuronally (Fig. 1A). These imaging analyses are challenging in living animals that can move out of the imaging area or out of focus. Applying anesthetics on the other hand will affect their neuronal activity. To restrict the movement of the animals while recording the neuronal GCaMP fluorescence of the whole animal, we established a microfluidic device that traps the nematodes (Fig. 1B and Fig. S1). This allows a non-invasive quantification of GCaMP fluorescence in living animals.

The workflow of the analysis using the microfluidic channels is depicted in Fig. 1C and starts with the synchronization of the nematodes. In this study, we analyzed young adult animals (4 days after hatching) as well as day-7- and day-10-old nematodes to reflect the progression of aging. Notably, animals expressing disease-associated aggregation-prone proteins display a reduced lifespan (median of 9 days for Aβ_1-42_ animals) and the time points of our analysis were chosen to account for the shortened lifespan of the disease models (Gallrein et al., 2021). For the GCaMP recordings, the nematodes were trapped into the microfluidic channels and their fluorescence intensity was recorded. The nematodes in the channels remained alive and were not harmed during this procedure. The animals were able to move with their head and tail, and lay eggs, suggesting that the physiological behavior was not impeded. Further, the experiment, starting from the loading of multiple nematodes into the microchannels to imaging the last one at the microscope, took less than 30 min.

To determine the level of Ca^2+^-based neuronal signaling in the nematodes, we quantified the fluorescence intensity as well as the protein levels that report on the abundance of the expressed GCaMP protein (Fig. 1C-E and Fig. S2) and then normalized the fluorescence intensity to the GCaMP protein levels (Fig. 1F). We like to point out that we normalized the fluorescence of neuronally expressed GCaMP (Fig. 1D) to GCaMP protein levels that were then normalized to GAPDH protein levels of whole-nematode lysates (Fig. S2 and Fig. 1E). This normalization was used for all subsequent analyses in this study, in order to account for variations in GCaMP expression levels in animals at different ages and in different strains. To further verify our observations, we also performed this analysis in *unc-13* mutant animals, which are impaired in synaptic vesicle fusion (Richmond et al., 1999); as expected, they showed a significant decrease in GCaMP intensity (Fig. 1F). As additional control, we applied the Ca^2+^-channel inhibitor nemadipine A to GCaMP-expressing control nematodes (Kwok et al., 2006). Nemadipine A-treated animals exhibited a significant decrease of GCaMP fluorescence (Fig. S3). We conclude that our experimental setup can be used to assess the average neuronal activity of living *C. elegans*.

### Neuronal function declines prior to the onset of significant Aβ_1-42_ aggregation

**Fig. 2.**
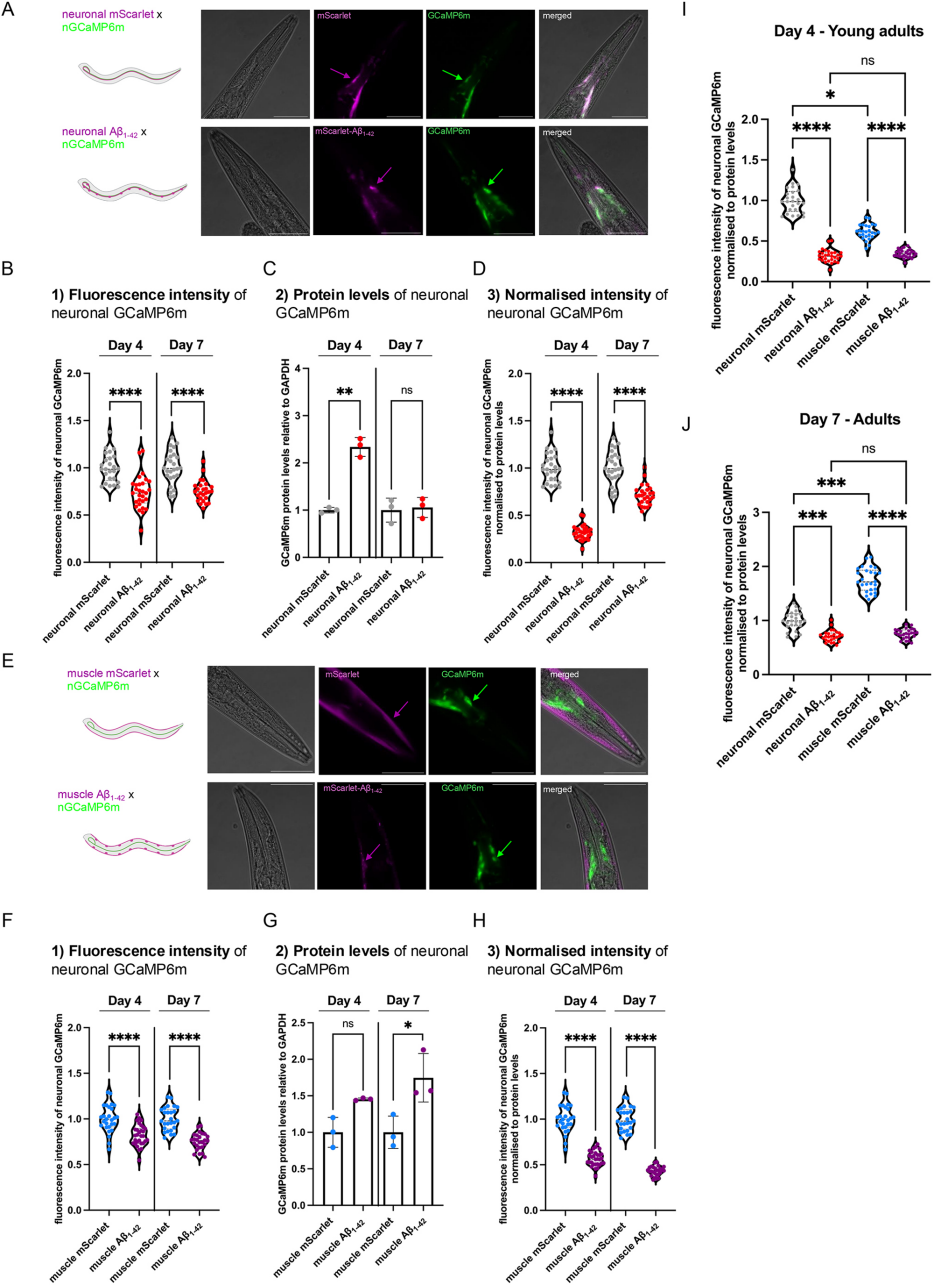
**Decrease of neuronal activity in Alzheimer's disease models precedes the accumulation of Aβ_1-42_ aggregates.** (A) Representative confocal fluorescent images of a young adult animal (day 4 of life) of the nmScarlet×nGCaMP6m cross (top panel) and of the cross nAβ_1-42_×nGCaMP6m (bottom panel). Shown are close-up images of the head region of the individual channels and of the merge. Images are 400-fold magnified, scale bars: 50 μm. (B) Violine dot plot of the average GCaMP6m fluorescence intensity of young adult animals (day 4 of life) and adult animals (day 7 of life) of nmScarlet and nAβ_1-42_ before normalization to GCaMP6m protein levels. GCaMP6m fluorescence intensities were measured in alive animals using microfluidic devices with a widefield fluorescence microscope and intensities were quantified using Fiji. Every dot represents the neuronal GCaMP6m fluorescence intensity of a single animal of nmScarlet (grey) and nAβ_1-42_ (red). *n*=3 and *N*=25-29 animals. Significance was assessed between age matched nmScarlet and nAβ_1-42_ animals (at age 4 and 7 days) by unpaired Student's *t*-test with Welch's correction (****P≤0.0001). (C) Quantification of GCaMP6m proteins levels by western blot of total protein lysates of young adult (day 4 of life) and adult animals (day 7 of life) of nmScarlet (grey) and nAβ_1-42_ (red) animals. Scatter dot plot shows quantification of GCaMP6m protein levels relative to whole nematode GAPDH from three independent cohorts. Significance was assessed between age matched nmScarlet and nAβ_1-42_ animals (at age 4 and 7 days) by unpaired Student's *t*-test with Welch's correction (ns, *P*>0.05; **P≤0.01). (D) Violine dot plot of the average GCaMP6m fluorescence intensity normalized to the GCaMP6m protein level of young adult animals (day 4 of life) and adult animals (day 7 of life) of the control strain expressing neuronal mScarlet (nmScarlet) and nAβ_1-42_. GCaMP6m intensities as plotted in B were multiplied with the ratio between nGCaMP6m protein levels of the nmScarlet×nGCaMP6m and nAβ_1-42_×nGCaMP6m cross for Day 4 and 7, respectively. Every dot represents the neuronal GCaMP6m fluorescence intensity normalized to whole nematode GCaMP6m protein levels of a single animal of nmScarlet (grey) and nAβ_1-42_ (red). *n*=3 and *N*=25-29 animals. Significance was assessed between age matched nmScarlet and nAβ_1-42_ animals (at age 4 and 7 days) by unpaired Student's *t*-test with Welch's correction (****P≤0.0001). (E) Representative confocal fluorescent images of a young adult animal (age 4 days) of the mmScarlet×nGCaMP6m cross (top panel) and of the mAβ_1-42_×nGCaMP6m cross (bottom panel). The mAβ_1-42_ strain as well as the respective mmScarlet control strain were crossed with the nGCaMP6 strain. Shown are close-up images of the head region of the individual channels and of the merge. Images are 400-fold magnified, scale bars: 50 μm. (F) Violine dot plot of the average GCaMP6m fluorescence intensity of young adult animals (day 4 of life) and adult animals (day 7 of life) of mmScarlet and mAβ_1-42_ before normalization to GCaMP6m protein levels. GCaMP6m intensities were measured in alive animals using microfluidic devices with a widefield fluorescence microscope and intensities were quantified using Fiji. Every dot represents the neuronal GCaMP6m fluorescence intensity of a single animal of mmScarlet (turquoise) and mAβ_1-42_ (purple). *n*=3-4 and *N*=26-30 animals. Significance was assessed between age matched mmScarlet and mAβ_1-42_ animals (at age 4 and 7 days) by unpaired Student's *t*-test with Welch's correction (****P≤0.0001). (G) Quantification of GCaMP6m proteins levels by western blot of total protein lysates of young adult (day 4 of life) and adult animals (day 7 of life) of mmScarlet (turquoise) and mAβ_1-42_ (purple) animals. Scatter dot plot shows quantification of GCaMP6m protein levels relative to whole nematode GAPDH from three independent cohorts. Significance was between age matched mmScarlet and mAβ_1-42_ animals (at age 4 and 7 days) by unpaired Student's *t*-test with Welch's correction (ns, *P*>0.05; *P<0.05). (H) Violine dot plot of the average GCaMP6m fluorescence intensity normalized to the GCaMP6m protein level of young adult animals (day 4 of life) and adult animals (day 7 of life) of the control strain expressing muscle mScarlet (mmScarlet) and mAβ_1-42_. GCaMP6m intensities were measured in alive animals using microfluidic devices with a widefield fluorescence microscope. GCaMP6m intensities as depicted in F were multiplied with the ratio between nGCaMP6m protein levels of the mmScarlet×nGCaMP6m cross and mAβ_1-42_×nGCaMP6m cross for Day 4 and 7, respectively. Every dot represents the neuronal GCaMP6m fluorescence intensity normalized to GCaMP6m protein levels of a single animal of mmScarlet (turquoise) and mAβ_1-42_ (purple). *n*=3-4 and *N*=26-30 animals. Significance was assessed between age matched mmScarlet and mAβ_1-42_ animals (at age 4 and 7 days) by unpaired Student's *t*-test with Welch's correction (****P≤0.0001). (I) Violine dot plot of the average GCaMP6m fluorescence intensity normalized to the GCaMP6m protein level of young adult animals (4 days old) of the nmScarlet, nAβ_1-42_, mmScarlet and mAβ_1-42_ strain. GCaMP6m intensities were measured in alive animals using microfluidic devices with a widefield fluorescence microscope and were multiplied with the ratio between nGCaMP6m protein levels of the nmScarlet×nGCaMP6m cross and nAβ_1-42_×nGCaMP6m/mmScarlet×nGCaMP6m / mAβ_1-42_×nGCaMP6m, respectively to allow comparison between tissues. Every dot represents the neuronal GCaMP6m fluorescence intensity normalized to GCaMP6m protein levels of a single animal of nmScarlet (grey), and nAβ_1-42_ (red), mmScarlet (turquoise) and mAβ_1-42_ (purple). *n*=3-4 and *N*=25-30 animals. The graph shows the same data sets depicted in individual graphs in D and H but compares the normalized GCaMP fluorescence in the different strains in young adult animals (day 4 of life). Significance was assessed by Kruskal–Wallis test with Dunn's test (****P≤0.0001; *P≤0.05; ns, *P*>0.05). (J) Violine dot plot of the average GCaMP6m fluorescence intensity normalized to the GCaMP6m protein level of adult animals (7 days old) of the nmScarlet, nAβ_1-42_, mmScarlet and mAβ_1-42_ strain. GCaMP6m intensities were measured in alive animals using microfluidic devices with a widefield fluorescence microscope and were multiplied with the ratio between nGCaMP6m protein levels of the nmScarlet×nGCaMP6m cross and nAβ_1-42_×nGCaMP6m/mmScarlet×nGCaMP6m/mAβ_1-42_×nGCaMP6m, respectively to allow comparison between tissues. Every dot represents the neuronal GCaMP6m fluorescence intensity normalized to GCaMP6m protein levels of a single animal of nmScarlet (grey), and nAβ_1-42_ (red), mmScarlet (turquoise) and mAβ_1-42_ (purple). *n*=3 and *N*=26 animals. The graph shows the same data sets depicted in individual graphs in D and H but compares the normalized GCaMP fluorescence in the different strains in adult animals (day 7 of life). Significance was assessed by Kruskal–Wallis test with Dunn's test (****P≤0.0001; ***P≤0.001; ns, *P*>0.05).

Aβ_1-42_-expressing animals show an age-dependent increase in Aβ_1-42_ aggregation, propagation and severe proteotoxicity (Gallrein et al., 2021). To correlate the expression and aggregation of Aβ_1-42_ with its effect on neuronal function, we crossed the pan-neuronal GCaMP (nGCaMP6m) reporter with our previously generated strain that expresses neuronal Aβ_1-42_ (nAβ) (Gallrein et al., 2021) or with control animals that only express the fluorescent protein mScarlet in the neurons (hereafter referred to as nmScarlet) (Gallrein et al., 2021) (Fig. 2A). We then measured the neuronal activity at two time points – at day 4 of life (young adults), when aggregation of Aβ_1-42_ begins; and day 7 of life (older adults), when animals show severe aggregation of Aβ_1-42_ (Fig. 2B-D; Fig. S5) – as has been demonstrated previously (Gallrein et al., 2021). Notably, compared to control, we observed a severe reduction in neuronal activity already in day-4-old Aβ_1-42_ animals (Fig. 2D,I). Day-7-old Aβ_1-42_ animals showed, as expected, a reduction in neuronal activity. At this age, Aβ_1-42_ aggregation affects all neurons, resulting in severe proteotoxicity as has been shown previously (Gallrein et al., 2021). Surprisingly, for Aβ_1-42_ animals, neuronal activity on day 4 was decreased compared to that of day 7 (Fig. 2D). At first glance, this may seem contradictory but can be explained with a survivorship bias, as the median lifespan of Aβ_1-42_ animals is 9 days (Gallrein et al., 2021). Thus, animals that are more severely affected by the aggregation and proteotoxicity of Aβ_1-42_ may have already died before day 7.

Next, we asked how Aβ_1-42_ expressed in a distal tissue affects neuronal activity. To address this, we made use of a muscle Aβ_1-42_ strain that expresses the Aβ_1-42_ peptide in body wall muscles (mAβ) (Gallrein et al., 2021). We crossed this strain as well as muscle mScarlet control nematodes (hereafter referred to as mmScarlet) with animals expressing neuronal GCaMP (Fig. 2E). Surprisingly, we observed similar reduction in neuronal activity when Aβ_1-42_ is expressed in muscle tissue, with this also already occurring in young adult animals (day 4 of life). Upon closer inspection, we noticed that the detrimental effect of Aβ_1-42_ on neuronal activity on day 7 of life is more pronounced when Aβ_1-42_ is expressed in muscle compared to neurons (Fig. 2H-J; Fig. S5). This could be a direct effect of propagated Aβ_1-42_ from muscle to neurons and/or transduced by a signaling cascade as aging progresses (van Oosten-Hawle, 2023). From these data, we conclude that neuronal activity decreased early in Aβ_1-42_-expressing animals and occurred before a significant accumulation of Aβ_1-42_ aggregates. In addition, neuronal activity was equally affected by distally expressed Aβ_1-42_.

### Generation of neuronal *C. elegans* Tau models based on the human Tau-0N4R isoform

**Fig. 3.**
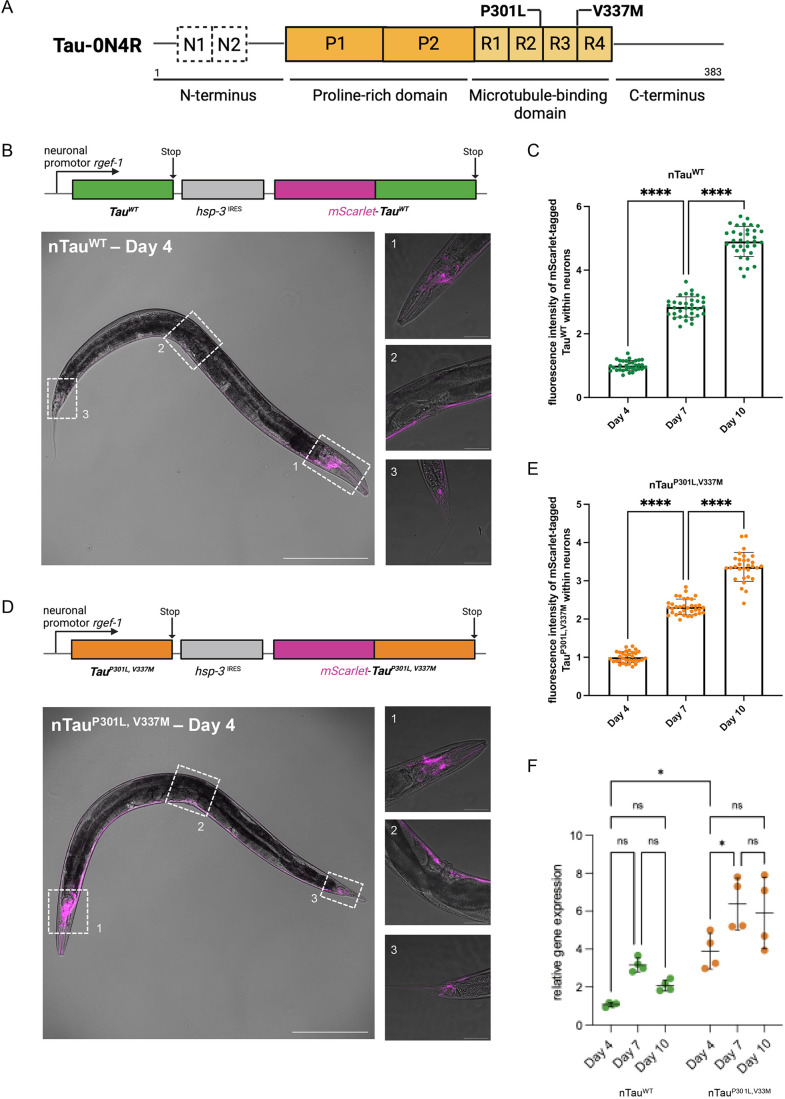
**Generation of a neuronal *C. elegans* Tau model with patient-derived mutations.** (A) Protein domain structure of the human Tau protein isoform 0N4R used for over-expression. The N-terminus of Tau-0N4R lacks the two N-terminal domains N1 and N2 (dashed squares) followed by a proline-rich domain, the four repeat domains (R1-R4) of the microtubule-binding domain and the C-terminus. The position of the two point-mutations Proline-to-Leucine at residue 301 (P301L) and Valine-to-Methionine at residue 337 (V337M) that are associated with frontotemporal dementia (FTD) are indicated and used to generate the mutant Tau strain. (B) Schematic representation of the neuronal Tau^WT^ (nTau^WT^) expression construct and corresponding confocal fluorescent images of a young adult (4 days old) animal. Untagged Tau^WT^ and mScarlet-tagged Tau^WT^ are pan-neuronally expressed driven by the *rgef-1* promotor. Cap-dependent translation leads to an overexpression of untagged Tau^WT^ and *hsp-3*^IRES^ cap-independent translation allows for sub-stoichiometric expression of mScarlet-Tau^WT^. Image on the left is 100-fold magnified (scale bar: 200 μm). Numbered images on the right show the respective close-ups of the head-, mid body and tail region. Close-up images on the right are 400-fold magnified (scale bars: 50 μm). (C) Quantification of mScarlet-Tau^WT^ fluorescence intensity during progression of aging of 4-, 7- and 10-day-old nematodes of the nTau^WT^ strain. Confocal fluorescent images of three cohorts of total 32-35 nematodes were recorded, and fluorescence intensities were quantified by Fiji. Data are displayed as mean fluorescence intensity±s.d. Significance was tested by one-way ANOVA+Bonferroni *post hoc* test (****P<0.0001). (D) Schematic representation of the neuronal Tau^P301L,V337M^ (nTau^P301L,V337M)^ construct and corresponding fluorescent confocal microscopy images of a young adult (day 4 of life) animal. The presented operon structure follows the same principle of expression as described in B. Image on the left is 100-fold magnified (scale bar: 200 μm). Numbered images on the right respective close-ups of the head-, mid body and tail region. Close-up images on the right are 400-fold magnified (scale bars: 50 μm). (E) Quantification of mScarlet-Tau^P301L,V337M^ fluorescence intensity during progression of aging of 4-, 7- and 10-day-old nematodes of nTau^P301L,V337M^ strain. Confocal fluorescent images of three cohorts of total 32-35 nematodes were recorded, and fluorescence intensities were quantified by Fiji. Data are displayed as mean fluorescence intensity±s.d. Significance was tested by one-way ANOVA+Bonferroni *post hoc* test (****P<0.0001). (F) qRT-PCR analysis of *Tau* gene expression. The graph depicts the relative human *Tau* gene expression of JKM150 (nTau^WT^, green) and JKM151 (Tau^P301L,V337M^, orange) on days 4, 7 and 10 of life. The expression data were normalized to the average expression of three reference genes (*act-1*, *lmn-1* and *eif-3C*). Three independent cohorts of ∼200 animals were analyzed. Shown is the mean±s.d. Ratio-paired *t*-test was performed. ns, non-significant; *P≤0.05.

The data obtained by using the Aβ_1-42_ model posed the question whether early decline of neuronal activity in AD animals is specific for Aβ_1-42_ or a general phenomenon in response to aggregating disease-associated amyloid proteins. We set out to perform the same analysis with the second amyloid protein that is associated with AD, Tau. Tau is a microtubule-binding, intracellular protein that can become hyperphosphorylated and form neurofibrillary tangles (Medeiros et al., 2011). Several point mutations have been identified in familial cases of AD that render Tau prone to aggregation (Wolfe, 2009). We generated strains that express the human 0N4R variant Tau in a non-modified form that is referred to as wild-type Tau (Tau^WT^) and a mutant Tau form harboring two patient-derived mutations, i.e. P301L and V337M (Tau^P301L,V337M^) in neurons (Fig. 3A). We have obtained one transgenic integrated line each for Tau^WT^ and Tau^P301L,V337M^. Additional lines may be needed in the future to, for example, control for a potential effect of the integration site of the transgene. Analogously to the Aβ_1-42_ model, Tau is expressed in an untagged and sub-stoichiometrically mScarlet-tagged manner by using an internal ribosome entry site (IRES) element for its synthesis (Fig. 3B,D; Fig. S6) (Gallrein et al., 2021). The sub-stoichiometric tagging with mScarlet limits perturbation of amyloid fibril formation by the fluorescent protein, yet, enables a visualization and assessment of Tau aggregation by fluorescence lifetime imaging microscopy (FLIM) in a non-invasive manner. Surprisingly, neuronal Tau levels increased with age from day 4 of life (young adult animals) up to day 10 of life (past their fertile period) for both Tau^WT^ and Tau^P301L,V337M^ (Fig. 3C,E; Fig. S7A), thereby differing from neuronal and muscular nAβ animals, which showed a decrease and no change in Aβ_1-42_ expression, respectively, with age (Fig. S7B,C). The increased abundance of Tau with aging is likely due to increased stability of the protein, as transcription levels for Tau^P301L,V337M^ were increased only between days 4 and 7, and did not increase further by day 10 and were unchanged for Tau^WT^ (Fig. 3F).

### Mutant Tau protein shows age-dependent aggregation and propagation

**Fig. 4.**
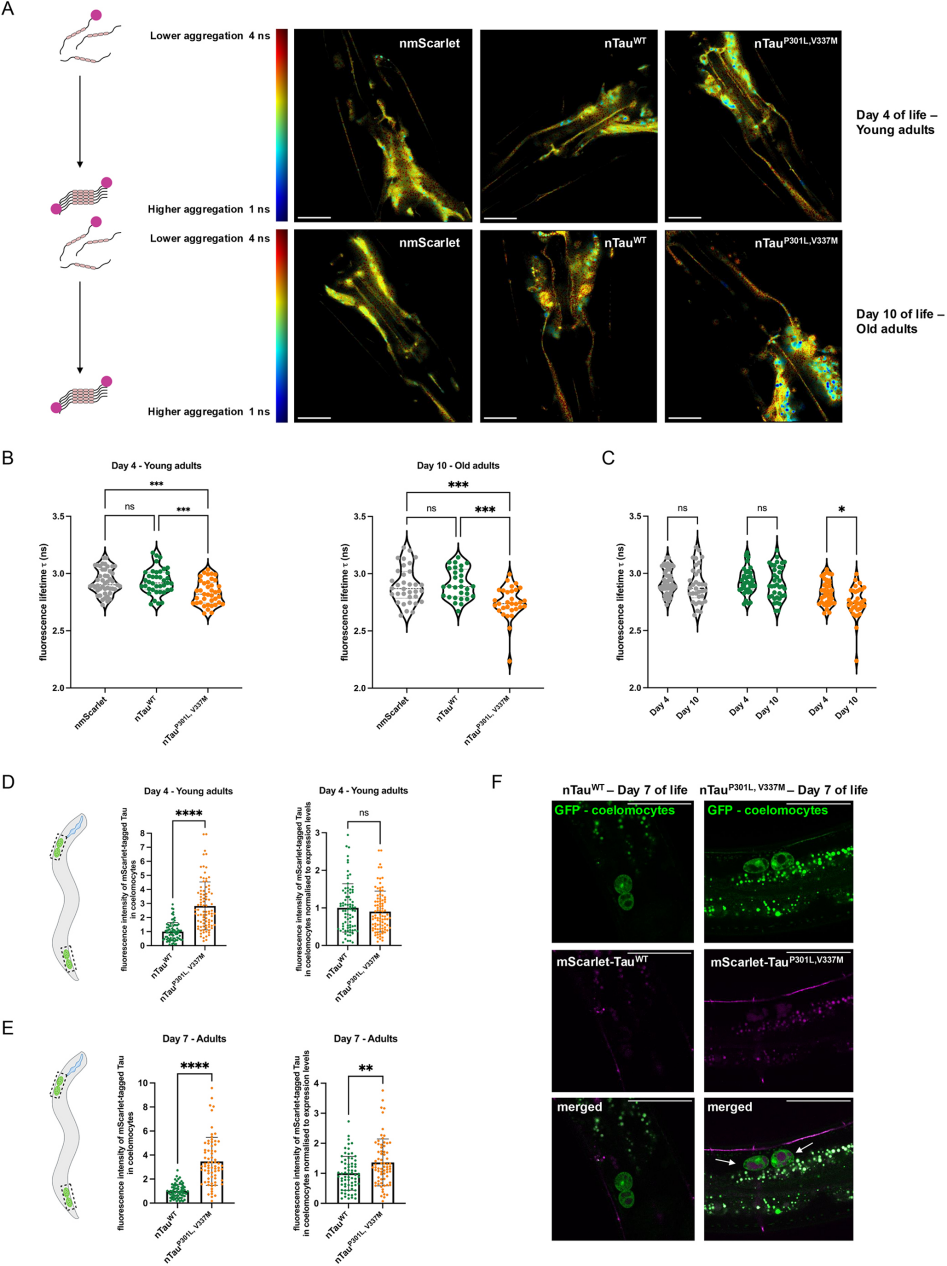
**Mutant Tau shows age-dependent aggregation and propagation.** (A) Representative TCSPC-FLIM images of nmScarlet, nTau^WT^ and nTau^P301L,V337M^. Head neurons of 4-day-old adult animals (Day 4 of life – Young adults (top row) and 4-day-old adult animals (Day 10 of life – Old adults (bottom row) were analyzed. Images depict the pixel-wise fluorescent lifetime (τ) merged with the fluorescent intensity. Fluorescence lifetime is false-colored: blue represents low fluorescent lifetimes (1 ns) showing regions with aggregated Tau and red represents high fluorescent lifetimes (4 ns) showing region with soluble Tau species that are schematically depicted on the left. Scale bars: 20 μm. (B) Violine dot plot of the average fluorescent lifetime (τ) of young adult animals (left) and old adult animals (right) of nmScarlet, nTau^WT^ and nTau^P301L,V337M^. Data displays average fluorescent lifetimes ±s.d. of nmScarlet (grey), nTau^WT^ (green) and nTau^P301L,V337M^ (orange). Every dot represents the average fluorescent lifetime for the head neurons of one single nematode. Three independent cohorts of 31-47 nematodes were analyzed. Significance was tested by one-way ANOVA+Bonferroni *post hoc* test for young adult animals and by Kruskal-Walls test with Dunn’s post-hoc test for old animals (ns, *P*>0.05; ***P≤0.001). (C) Violine dot plot of the average fluorescent lifetime (τ) of young adult animals (4 days old) and old adult animals (10 days old) of nmScarlet, nTau^WT^ and nTau^P301L,V337M^. The graph shows the combined data sets depicted in individual graphs of (B). Data displays average fluorescent lifetimes±s.d. of nmScarlet (grey), nTau^WT^ (green) and nTau^P301L,V337M^ (orange). Every dot represents the average fluorescent lifetime for the head neurons of one single nematode. Three independent cohorts of 31-47 animals were analyzed. Significance was tested by two-way ANOVA+Bonferroni *post hoc* test (ns, *P*>0.05; *P≤0.05). (D) Scatter dot plot of the average Tau levels in the coelomocytes of nTau^WT^ and nTau^P301L,V337M^ young adult animals (left graph) as well as normalized to the Tau expression levels (right graph). For coelomocyte identification nTau^WT^ and nTau^P301L,V337M^ were crossed with the *C. elegans* strain ZIM1048 that expresses GFP in coelomocytes and is used as marker for coelomocytes. We excluded day 10 analyses due to the increased autofluorescence at that age. Tau fluorescence levels were quantified in the head and the tail coelomocytes by Fiji analysis of confocal images. Every dot represents the level of Tau in head or the tail coelomocytes for nTau^WT^ (green) and nTau^P301L,V337M^ (orange). Fluorescence intensities were normalized to day 4 of the signals of nTau^WT^. Three cohorts of 32-38 nematodes were analyzed. Significance was assessed by Mann–Whitney test (ns, *P*>0.05; ****P≤0.0001). (E) Scatter dot plot of the average Tau levels in the coelomocytes of nTau^WT^ and nTau^P301L,V337M^ adult animals (left graph) as well as normalized to the Tau expression levels (right graph). Tau fluorescence levels were quantified in the head and the tail coelomocytes by Fiji analysis of confocal images. Every dot represents the level of Tau in head or the tail coelomocytes for nTau^WT^ (green) and nTau^P301L,V337M^ (orange). Fluorescence intensities were normalized to day 7 of the signals of nTau^WT^. Three cohorts of 37-38 nematodes were analyzed. Significance was assessed by Mann–Whitney test (**P≤0.01; ****P≤0.0001). (F) Confocal fluorescent images of nTau^WT^ (left) and nTau^P301L,V337M^ (right) adult animals (day 7 of life) crossed with the coelomocyte marker (*unc-122p::gfp*) strain ZIM1048. Scale bars: 50 μm. Arrows in the merged panel of Tau^P301L,V337M^ point to coelomocytes that show incorporated magenta, fluorescent material representing mScarlet-tagged Tau^P301L,V337M^.

Next, we analyzed the aggregation of Tau using fluorescence-lifetime imaging microscopy (FLIM) (Fig. 4A). FLIM is an advanced microscopy technique capable of providing a deeper understanding of the molecular environment of a fluorophore. Amyloid formation leads to quenching of the mScarlet fluorophores that are fused to Tau, which reduces the fluorescence lifetime (τ) of the fluorophore (Chen et al., 2017). Thus, the fluorescence lifetime can be used as readout for the aggregation of Tau (Pigazzini et al., 2020). The control (nmScarlet) and Tau^WT^ were soluble, whereas Tau^P301L,V337M^ aggregated already in young adult animals (Fig. 4B,C). The aggregation of Tau^P301L,V337M^ is also depicted by the color change in false-colored images, in which blue areas indicate regions of low τ values (i.e. aggregates) and are reflected by the overall decreased τ values of the head neurons (Fig. 4A). We further observed that aggregation of mutant Tau (Tau^P301L,V337M^) increased with age, while that of control and Tau^WT^ remained soluble (Fig. 4C). These results highlight that the increased abundance observed for both Tau^WT^ and Tau^P301L,V337M^ with aging (Fig. 3C,E) is not necessarily leading to increased aggregation propensity and that, instead, the P301L and V337M mutations cause Tau aggregation. Interestingly, we did not identify a specific neuron or neuron type that shows early onset of Tau aggregation in mutant Tau lines, as it has previously been observed for Aβ_1-42_, whose aggregation starts in in a subset of neurons of the anterior head ganglion, i.e. the six IL2 neurons (Fig. 4A) (Gallrein et al., 2021).

A hallmark of several neurodegenerative diseases, such as AD, is the spreading of disease-associated amyloid proteins. We, hence, analyzed spreading of Tau^P301L,V337M^ to other tissues. To quantify this, we measured Tau protein levels in coelomocytes, scavenger leucocytes that take up extracellular material from the body cavity. Of note, only proteins that are released by neurons as part of the spreading and propagation of Tau enter the extracellular space and get endocytosed by coelomocytes, where they can be subjected to lysosomal degradation. We crossed the Tau strains with the ZIM1048 strain that expresses GFP in coelomocytes (Nichols et al., 2017) that served as a marker to easily identify them and then quantify Tau levels in coelomocytes. On days 4 and 7 of life, we detected a 2.8-fold (day 4) or even 3.5-fold (day 7) increase of mutant Tau compared to Tau^WT^ in coelomocytes (Fig. 4D-F). When we normalized Tau abundance in coelomocytes to Tau expression levels, it became clear that spreading of Tau^P301L,V337M^ was still significantly higher than that of Tau^WT^ – yet, only in older animals (Fig. 4D,E, right plots), an age when we also observed strong aggregation of Tau^P301L,V337M^ by FLIM (Fig. 4A-C).

### Aggregation of Tau is associated with severe organismal defects

**Fig. 5.**
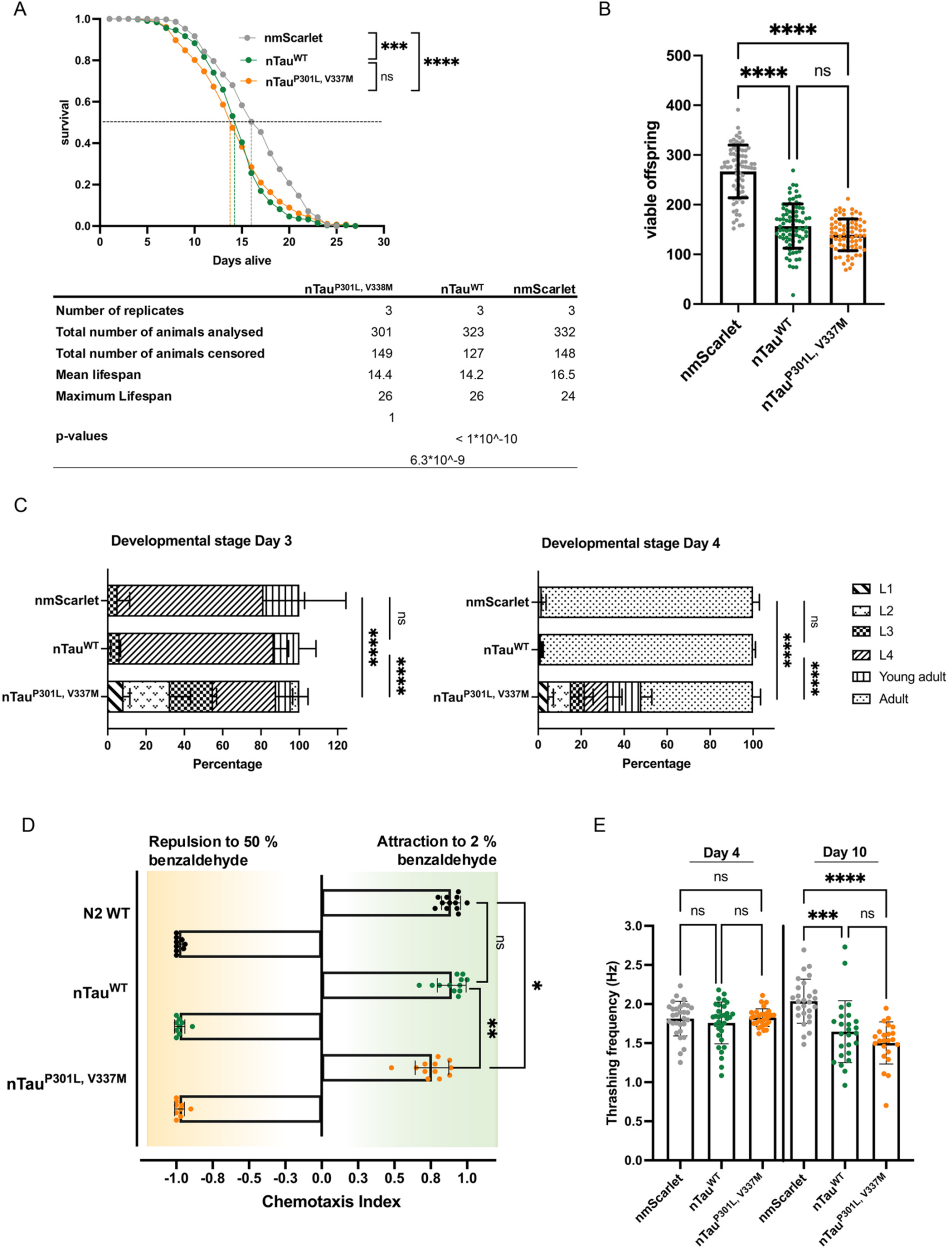
**Aggregation of Tau is associated with severe organismal defects.** (A) Assessment of the lifespan of nmScarlet, nTau^WT^ and nTau^P301L,V337M^ animals. Graph shows the cumulative survival probability (survival) versus age (days alive) of nmScarlet (grey), nTau^WT^ (green) and nTau^P301L,V337M^ (orange). Three independent cohorts of 150-180 nematodes each were analyzed, and significance was tested by log-rank test using Oasis2 online tool (ns, *P*>0.05; ***P≤0.001, ****P≤0.0001). The table below summarizes all parameter. (B) Fecundity analysis of nmScarlet, nTau^WT^ and nTau^P301L,V337M^ animals. Scatter dot plot displays average number of viable offspring of nmScarlet (grey), nTau^WT^ (green) and nTau^P301L,V337M^ (orange). Every dot represents the absolute number of viable offspring of a single nematode. Significance was assessed by one-way ANOVA+Bonferroni *post hoc* test (ns, *P*>0.05; ****P≤0.0001). Three independent cohorts of 74-83 nematodes were tested. (C) Developmental assay of nmScarlet, nTau^WT^ and nTau^P301L,V337M^ animals. Graph displays percentage of developmental stages of the nematodes within a population at day 3 (left) and day 4 (right) of life. Three independent cohorts of 40-60 nematodes each were analyzed. Kruskal–Wallis test was employed to test significance between fractions of L4 animals (left) and adult animals (right) (****P≤0.0001). (D) Assessment of chemotaxis towards 50% (repulsion) and 2% (attraction) benzaldehyde of nTau^WT^ and nTau^P301L,V337M^ animals. Scatter plot displays chemotaxis index of nTau^WT^ (green) and nTau^P301L,V337M^ (orange) whereas each dot represents the calculated chemotaxis index for one technical replicate. Three to four independent cohorts of each 200-300 nematodes (young adults) were tested, divided into three technical replicates. Significance was assessed by Kruskal–Wallis test with Dunn's test (ns, *P*>0.05; *P≤0.05; **P≤0.01). (E) Scatter dot plot of the thrashing capability of nmScarlet (grey), nTau^WT^ (green) and nTau^P301L,V337M^ (orange) animals at day 4 (left) and day 10 (right) of life. Every dot represents the trashing frequency of a single nematode. Three cohorts of 8-15 nematodes per strain were recorded for 20 s and thrashing frequency was analyzed from the recorded videos for each animal individually. Significance for days 4 and 10 was assessed by Kruskal–Wallis test with Dunn's test (ns, *P*>0.05; ***P≤0.001; ****P≤0.0001).

Next, we performed a phenotypic analysis of the new Tau strains to comprehensively characterize them and to assess the proteotoxicity of Tau. Tau^P301L,V337M^ expression led to a significantly reduced lifespan with a median lifespan of 14.2 days in comparison to a median lifespan of 16.5 days for nmScarlet control (Fig. 5A; Fig. S10). Further, we assessed several physiological parameters that report on the fitness of animals. We observed a significant 48% (*P*≤0.0001) reduction in the number of progenies, from a mean number of 267 offspring for nmScarlet control animals to 139 for Tau^P301L,V337M^ animals (Fig. 5B). Additionally, development of mutant Tau animals was severely delayed. This was already apparent on day 3 of life and even more on day 4 of life, when about 48% of all mutant Tau animals were still at larval stage while control animals had all reached adulthood (Fig. 5C). Alongside this, a chemotaxis assay revealed that mutant Tau animals were impaired to respond to the volatile attractant benzaldehyde (2%), with a mean chemotaxis index of 0.76 compared to N2 wild-type and Tau^WT^ animals, thereby, showing a mean chemotaxis index of 0.89 and 0.9, respectively (Fig. 5D). Finally, motility, as assessed by thrashing frequency in liquid medium, was reduced to a mean thrashing frequency of 1.5 Hz in older mutant Tau animals compared to a thrashing frequency of 2 Hz in nmScarlet controls (Fig. 5E). Notably, we also observed a reduction of mean thrashing frequency in the Tau^WT^ strain, which was unexpected as Tau^WT^ does not aggregate (Fig. 4A-C). We noticed that Tau^WT^ elicited its detrimental effect only in assays assessing nematodes over longer periods (i.e. up to day 25 of life) or fecundity (i.e. up to day 8 of life), but did not observe defects regarding development on days 3 and 4 of life (Fig. 5C), chemotaxis on day 4 of life (Fig. 5D) or motility on day 4 of life (Fig. 5E). Defects in motility were, again, only detected on day 10 of life (Fig. 5E). Thus, expression of Tau^WT^ exerted a negative and aggregation-independent effect on the physiology of older nematodes.

We set out to further study the neuronal ectopic expression of human Tau^WT^ in *C. elegans*. The endogenous *C. elegans* Tau ortholog PTL-1 is expressed in several neurons – most strongly in ALM, AVM and PLM touch neurons – and is required for neuronal integrity (Chew et al., 2013; Kraemer et al., 2003). PTL-1A and PTL-1B are two isoforms that show <50% sequence identity with human Tau (44% and 47%, respectively), which is, however, restricted to the microtubule-binding domains (Fig. S8B) (Goedert et al., 1996). Mutation of *ptl-1* leads to neuronal morphological defects and a shortened lifespan (Chew et al., 2013). We could reproduce these data and observed a reduction in the lifespan of *ptl-1* mutant (*ok621*; Fig. S8A). It has previously been shown that the shortened lifespan of *ptl-1* mutant animals can be rescued by ectopic expression of *ptl-1* (Chew et al., 2013). However, when we crossed the *ptl-1* mutant with our newly generated neuronal human Tau^WT^ strain, we observed an even further reduction in lifespan from a median lifespan of 15.4 days (*ptl-1* deletion mutant) and 14.2 (pan-neuronal overexpression human Tau^WT^) to 11.5 days for the cross of *ptl-1* mutant and neuronal human Tau^WT^ (Fig. S8A). These data suggest that human Tau differs from its *C. elegans* homolog and cannot substitute for its loss. Thus, the cross of *ptl-1* mutant and human Tau^WT^ is deficient for the endogenous Tau protein and expresses a protein that is dominantly negative on the physiology of the nematode. Importantly, Tau^WT^ does not exert its toxicity by aggregation (Fig. 4A-C). We, thus, argue that Tau^WT^ is an unsuitable control to assess the aggregation-associated toxicity of Tau^P301L,V337M^ and that, instead, the mScarlet-expressing strain should be used as control for the toxic gain-of-function of amyloid fibril-forming Tau^P301L,V337M^.

Tau^P301L,V337M^, however, showed a proteotoxic effect in all behavioral assays. The defects in neuronal function as reported in the chemotaxis assay, in which Tau^P301L,V337M^ animals were impaired to respond to an attractant, led us to hypothesize that mutant Tau animals are compromised in their neuronal activity.

### Neuronal function declines prior to the onset of significant Tau aggregation

**Fig. 6.**
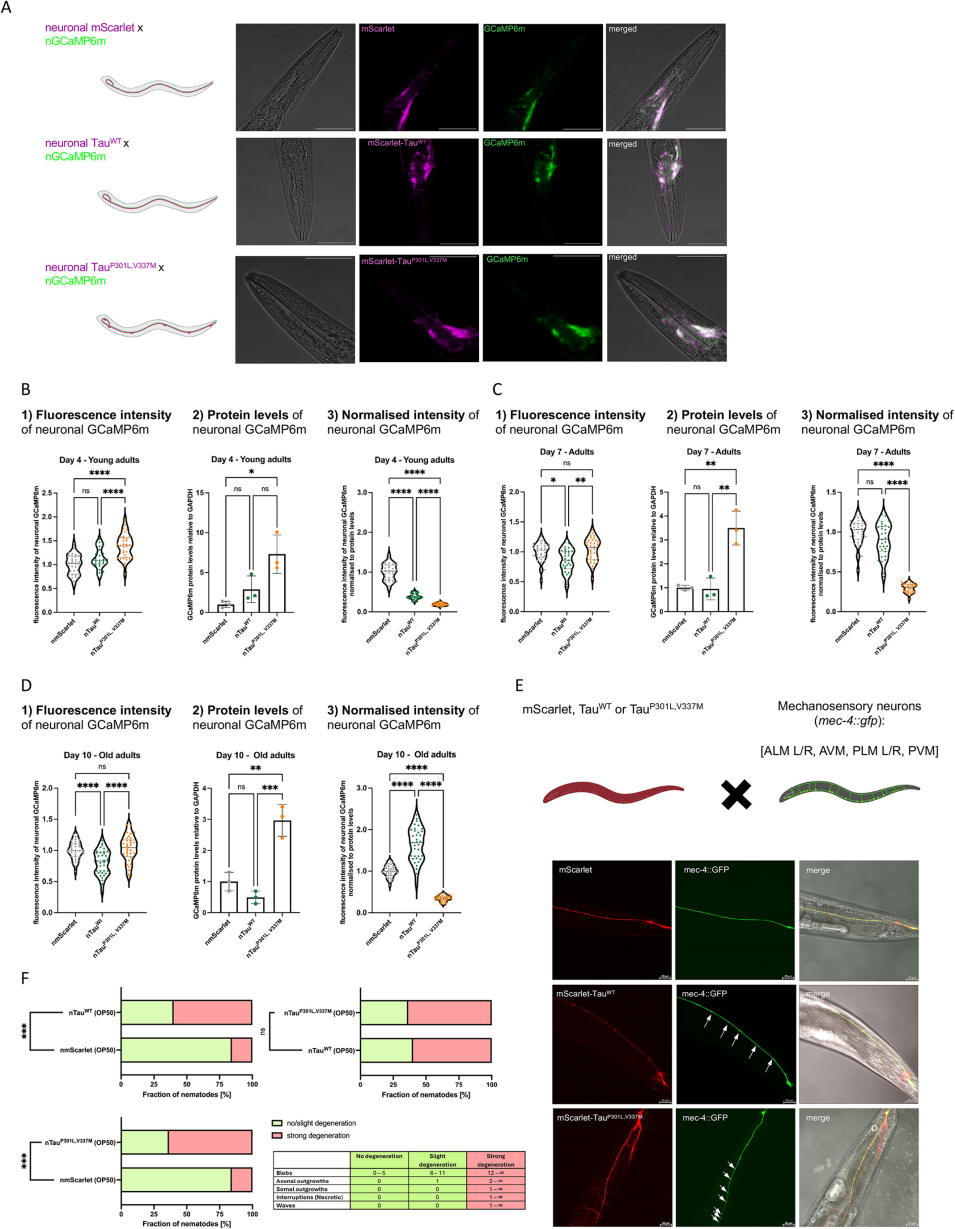
**Loss of neuronal function precedes Tau aggregation.** (A) Representative confocal fluorescent images of a young adult animal (day 4 of life) of the nmScarlet×nGCaMP6m, nTau^WT^×nGCaMP6m, and nTau^P301L,V337M^×nGCaMP6m cross from left to right. The Tau-expressing strains as well as the nmScarlet control strain were crossed with the nGCaMP6 strain. Shown are close-up images of the head region. Images are 400-fold magnified, scale bars: 50 μm. (B) (1) Violine dot plot of the average GCaMP6m fluorescence intensity of young adult animals (4 days old) of the control strain nmScarlet, nTau^WT^ and nTau^P301L,V337M^ before normalization to GCaMP6m protein levels. GCaMP6m intensities were measured in alive animals using microfluidic devices with a widefield fluorescence microscope and intensities were quantified using Fiji. Every dot represents the neuronal GCaMP6m fluorescence intensity of a single animal of nmScarlet (grey) and nTau^WT^ (green) and nTau^P301L,V337M^ (orange). *n*=5 and *N*=31-34 animals. Significance was assessed by one-way ANOVA+Bonferroni *post hoc* test (ns, *P*>0.05; ****P≤0.0001. (2) Quantification of GCaMP6m proteins levels by western blot of total protein lysates of young adult (day 4 of life) of nmScarlet (grey), nTau^WT^ (green) and nTau^P301L,V337M^ (orange) animals. Scatter dot plot shows quantification of GCaMP6m protein levels relative to whole nematode GAPDH of three independent cohorts. Significance was assessed by one-way ANOVA+Bonferroni *post hoc* test (ns, *P*>0.05; *P<0.05). (3) Violine dot plot of the average GCaMP6m fluorescence intensity normalized to the GCaMP6m protein level of young adult animals (day 4 of life) of the control strain nmScarlet, nTau^WT^ and nTau^P301L,V337M^. GCaMP6m intensities were measured in alive animals using microfluidic devices with a widefield fluorescence microscope. GCaMP6m intensities were multiplied with the ratio between nGCaMP6m protein levels of the nmScarlet×nGCaMP6m cross and nTau^WT^×nGCaMP6m cross/nTau^P301L,V337M^×nGCaMP6m, respectively. GCaMP6m protein quantification is shown in 2) and the corresponding full-length western blots in Fig. S9. Every dot represents the neuronal GCaMP6m fluorescence intensity normalized to GCaMP6m protein levels of a single animal of nmScarlet (grey) and nTau^WT^ (green) and nTau^P301L,V337M^ (orange). *n*=5 and *N*=31-34 animals. Significance was assessed by one-way ANOVA+Bonferroni *post hoc* test (****P≤0.0001). (C) (1) Violine dot plot of the average GCaMP6m fluorescence intensity of adult animals (day 7 of life) of the control strain nmScarlet, nTau^WT^ and nTau^P301L,V337M^ before normalization to GCaMP6m protein levels. GCaMP6m intensities were measured in alive animals using microfluidic devices with a widefield fluorescence microscope and intensities were quantified using Fiji. Every dot represents the neuronal GCaMP6m fluorescence intensity normalized to GCaMP6m protein levels of a single animal of nmScarlet (grey) and nTau^WT^ (green) and nTau^P301L,V337M^ (orange). *n*=4-5 and *N*=31-35 animals. Significance was assessed by Kruskal–Wallis test with Dunn's test (ns, *P*>0.05; *P≤0.05; **P≤0.01). (2) Quantification of GCaMP6m proteins levels by western blot of total protein lysates of adult (day 7 of life) of nmScarlet (grey), nTau^WT^ (green) and nTau^P301L,V337M^ (orange) animals. Scatter dot plot shows quantification of GCaMP6m protein levels relative to GAPDH from three independent cohorts. Significance was assessed by one-way ANOVA+Bonferroni *post hoc* test (ns, *P*>0.05; **P≤0.01). (3) Violine dot plot of the average GCaMP6m fluorescence intensity normalized to the GCaMP6m protein level of adult animals (day 7 of life) of the control strain nmScarlet, nTau^WT^ and nTau^P301L,V337M^. GCaMP6m intensities were measured in alive animals using microfluidic devices with a widefield fluorescence microscope. GCaMP6m intensities were multiplied with the ratio between nGCaMP6m protein levels of the nmScarlet×nGCaMP6m cross and nTau^WT^×nGCaMP6m cross/nTau^P301L,V337M^×nGCaMP6m, respectively. GCaMP6m protein quantification is shown in (2) and the corresponding full-length western blots in Fig. S9. Every dot represents the neuronal GCaMP6m fluorescence intensity normalized to GCaMP6m protein levels of a single animal of nmScarlet (grey) and nTau^WT^ (green) and nTau^P301L,V337M^ (orange). *n*=4-5 and *N*=31-35 animals. Significance was assessed by Kruskal–Wallis test with Dunn's test (ns, *P*>0.05; ****P≤0.0001). (D) (1) Violine dot plot of the average GCaMP6m fluorescence intensity of old adult animals (10 days old) of the control strain nmScarlet, nTau^WT^ and nTau^P301L,V337M^ before normalization to GCaMP6m protein levels. GCaMP6m intensities were measured in alive animals using microfluidic devices with a widefield fluorescence microscope and intensities were quantified using Fiji. Every dot represents the neuronal GCaMP6m fluorescence intensity normalized to GCaMP6m protein levels of a single animal of nmScarlet (grey) and nTau^WT^ (green) and nTau^P301L,V337M^ (orange). *n*=4-5 and *N*=34-37 animals. Significance was assessed by one-way ANOVA+Bonferroni *post hoc* test (ns, *P*>0.05; ****P≤0.0001). (2) Quantification of GCaMP6m proteins levels by western blot of total protein lysates of nmScarlet (grey), nTau^WT^ (green) and nTau^P301L,V337M^ (orange) old-adult animals. Scatter dot plot shows quantification of GCaMP6m protein levels relative to GAPDH from three independent cohorts. Significance was assessed by one-way ANOVA+Bonferroni *post hoc* test (ns, *P*>0.05; **P≤0.01; ***P≤0.001). (3) Violine dot plot of the average GCaMP6m fluorescence intensity normalized to the GCaMP6m protein level of old adults of the control strain nmScarlet, nTau^WT^ and nTau^P301L,V337M^. GCaMP6m intensities were measured in alive animals using microfluidic devices with a wide-field fluorescence microscope. GCaMP6m intensities were multiplied with the ratio between nGCaMP6m protein levels of the nmScarlet×nGCaMP6m cross and nTau^WT^×nGCaMP6m cross/nTau^P301L,V337M^×nGCaMP6m, respectively. GCaMP6m protein quantification is shown in (2) and the corresponding full-length western blots in Fig. S9. Every dot represents the neuronal GCaMP6m fluorescence intensity normalized to GCaMP6m protein levels of a single animal of nmScarlet (grey) and nTau^WT^ (green) and nTau^P301L,V337M^ (orange). *n*=4-5 and *N*=34-37 animals. Significance was assessed by one-way ANOVA+Bonferroni *post hoc* test (****P≤0.0001). (E) Analysis of neurodegeneration of mechanosensory neurons as assessed by neuronal integrity of control animals (nmScarlet) and those expressing Tau^WT^ and Tau^P301L,V337M^ using *mec-4::gfp* as reporter. The crossing scheme is indicated above the representative images of the generated crosses, depicting individual and merged images of 7-day-old animals. signs of neurodegeneration, i.e. protrusions and neurite swellings were observed for Tau^WT^ and Tau^P301L,V337M^ animals and are indicated by arrows. Images are 400-fold magnified, scale bars: 20 µm. (F) Quantification of neurodegeneration. Animals were divided into two groups: (i) no or slight degeneration of animals, showing only up to ten swellings (blebs) and a maximum of one axonal outgrowth (underlaid in green); and (ii) strong degeneration of animals, showing more than ten blebs, more axonal or somatic outgrowths and interrupted axons or wavy structures of their mechanosensory neurons (underlaid in green). Bar graph at top left: comparison between nTau^WT^ (*n*=30) and nmScarlet (*n*=32). Bar graph at top right: comparison between Tau^P301L,V337M^ (*n*=33) and nTau^WT^ (*n*=30). Bar graph at bottom left: comparison between Tau^P301L,V337M^ (*n*=33) and nmScarlet (*n*=32). Significance was assessed by Fisher's exact test (ns, *P*>0.05; ***P≤0.001).

To correlate aggregation of Tau^P301L,V337M^ with neuronal activity, we crossed the Tau lines with the GCaMP reporter and quantified GCaMP fluorescence analogous to the analyses carried out for Aβ_1-42_ (Fig. 6A-D; Fig. S9). Of note, GCaMP expression did not affect the age-associated increase of aggregation propensity of Tau^P301L,V337M^ but we noticed an effect on Tau^WT^, i.e. an increase of fluorescence lifetimes on day 4 of life and a reduction of fluorescence lifetimes on day 10 of life compared to those for nmScarlet (Fig. S4). Already in young adult animals (day 4 of life), we observed significant reduction of relative GCaMP intensity in Tau^P301L,V337M^, which intensified during aging (days 7 and 10 of life) (Fig. 6B-D). We, therefore, conclude, that expression of the highly aggregation-prone Tau^P301L,V337M^ results in severe reduction of neuronal activity already in young adult animals, similar to expression of Aβ_1-42_. Importantly, the significant defects in neuronal activity occurred early (young adult, day 4 of life) and preceded the continued build-up of Tau^P301L,V337M^ aggregates with age (Fig. 4A-C). Notably, we also observed reduced GCaMP intensities upon expression of Tau^WT^ in young adult animals (day 4 of life) – which, however, increased with age (Fig. 6B-D) – further supporting our observation that ectopic Tau^WT^ expression alters the physiology of the animal. We then asked the question whether the loss of neuronal activity is associated with neurodegeneration. To monitor neuronal integrity, we crossed animals expressing neuronal mScarlet, Tau^WT^ or Tau^P301L,V337M^ with a strain expressing GFP in the mechanosensory neurons (*mec-4::gfp*) and visualized neuronal morphology in older adult animals (day 7 of life) (Fig. 6E). While we did not observe morphological aberrations in mScarlet-expressing animals, we did detect neuronal swellings and protrusions in animals expressing Tau^P301L,V337M^ (Fig. 6E,F). This neurodegeneration is in line with our observed loss of neuronal activity and function of Tau^P301L,V337M^ animals (Figs 5D, 6A-D). We also observed defects in neuronal morphology in day-7-old Tau^WT^ animals (Fig. 6E,F), an unsurprising finding, as we also noticed physiological defects in older Tau^WT^ adult animals (Fig. 5).

## DISCUSSION

Amyloid formation is a hallmark of several neurodegenerative diseases, such as AD, and characterized by the deposition of Aβ plaques and Tau neurofibrillary tangles within different areas of the brain. We have recently established a *C. elegans* Aβ AD model that recapitulates the pathological features of AD, such as aggregation, spreading and propagation of Aβ, as well as severe physiological defects, such as shortened lifespan, reduced number of progenies, and impaired motility (Gallrein et al., 2021).

In this current study, we established a new *C. elegans* Tau AD model that expresses human wild-type or mutant Tau (Tau^WT^ or Tau^P301L,V337M^, respectively) to complement the Aβ AD model. Previously published *C. elegans* Tau models relied mainly on the expression of untagged Tau or used a stoichiometric GFP-Tau fusion (Kraemer et al., 2003; Aquino Nunez et al., 2022; Fatouros et al., 2012; Morelli et al., 2018; Pir et al., 2016; Miyasaka et al., 2005). Expression of untagged Tau maintains the characteristics of different Tau variants, yet requires extraction and fractionation procedures to assess the aggregation status of Tau (Kraemer et al., 2003; Aquino Nunez et al., 2022; Fatouros et al., 2012; Morelli et al., 2018). Here, we used a sub-stoichiometric labelling approach that we have used previously for Aβ (Gallrein et al., 2021), in order to obtain transgenic Tau lines that express an excess of untagged Tau to preserve the properties of the protein as well as a sub-stoichiometrically mScarlet-tagged Tau to monitor its aggregation in living animals using FLIM. We observed increased aggregation of Tau^P301L,V337M^ with aging. However, we did not detect a specific onset of aggregation – as previously been detected for Aβ, for which aggregation starts in the cholinergic IL2 neurons (Gallrein et al., 2021) – suggesting that neuronal vulnerability to the proteotoxicity of amyloid proteins differs. For example, this could be due to the neuron-specific composition of the proteostasis network or caused by differences in interaction partners with the amyloid proteins.

The expression and aggregation of Tau^P301L,V337M^ elicited multiple disease-associated phenotypes, such as reduced lifespan, delayed development as well as impaired fecundity and motility. We were able to further demonstrate loss of neuronal activity and integrity that manifests as neurite protrusions and swellings and, thus, further support the clear connection between aggregation propensity, propagation and neurodegeneration of mutant Tau. While proteotoxicity of mutant Tau has been observed previously (Kraemer et al., 2003; Aquino Nunez et al., 2022; Fatouros et al., 2012; Morelli et al., 2018; Pir et al., 2016; Miyasaka et al., 2005), our study comprehensively analyzed the aggregation of Tau^P301L,V337M^ and its detrimental effect on neuronal activity with aging. Of note, expression of human Tau^WT^ negatively affected lifespan, fecundity and motility of animals, despite the absence of aggregation of Tau^WT^ (Fig. 4A-C), corroborated by previous findings (Chew et al., 2013; Pir et al., 2016). We cannot rule out that human Tau^WT^ binds to microtubules in *C. elegans*, thus, interfering with the physiology of the animal.

By using the Aβ and Tau^P301L,V337M^ *C. elegans* AD models, we show that neuronal activity declined already in young adult animals and, thus, early in the AD pathology for both Aβ and mutant Tau. The loss of neuronal activity, as assessed by GCaMP fluorescence recording, manifests also in impaired neuronal function that is reflected in chemotactic defects. Although aggregation of Aβ (Gallrein et al., 2021) and mutant Tau further increases as the animals age, neuronal activity is already at its minimum for young adult animals of these AD strains. We, thus, speculate that early loss of neuronal function is a common hallmark of neurodegenerative diseases.

In contrast to studies conducted in murine APP/Aβ models, we did not detect early hyperactivity preceding the degeneration of neurons of our Aβ_1-42_ and Tau^P301L,V337M^ animals. However, we observed increased neuronal activity of day-10-old nematodes that express Tau^WT^ and that also show neuronal morphological aberrations. We have, so far, only analyzed mechanosensory neurons, and the activity and integrity of selected neurons or neuronal circuits might differ. To address this caveat, the GCaMP Ca^2+^ sensor and neuronal fluorescence marker could, in future, be expressed in neuronal subpopulations to elucidate potential differences of neurons regarding their susceptibility towards proteotoxic stress.

Surprisingly, we observed that the expression and aggregation of Aβ in muscle tissue had a similar detrimental effect on neuronal activity as its expression in neurons. This poses the question of how the proteotoxic stress in muscle tissue affects neuronal activity. Aβ can propagate from cell to cell and, hence, Aβ expression and aggregation in muscles can lead to spreading of Aβ into neurons (Gallrein et al., 2021). Alternatively, signaling pathways could relay the proteotoxicity of the muscle tissue to the neurons. Trans-cellular signaling of proteotoxicity and capacity of molecular chaperones has been observed before but mainly for signaling from neurons to peripheral tissue (van Oosten-Hawle et al., 2013; Taylor et al., 2014). Much less is known about a retrograde signaling from peripheral tissues to the nervous system. Such a signaling circuit from muscle to neurons has been observed in *Drosophila*. For example, muscle FOXO−4E-BP signaling leads to decreased feeding behavior, resulting in reduced insulin release that, in turn, promotes FOXO−4E-BP activity in other tissues to mitigate systemic aging (Demontis and Perrimon, 2010). It is possible that the reduced neuronal activity in response to Aβ aggregation in muscle tissue is communicated by a signaling pathway that may also involve neuromuscular junctions that provide a direct contact between muscle cells and neurons.

Furthermore, in this study, we have advanced the methodological portfolio to assess neuronal activity in living nematodes in a non-invasive manner by using a microfluidic device. Neuronal GCaMP recordings require immobilization of the animals without using anesthetics. Thus far, a myriad of microfluidic tools have been introduced that aim at supporting the investigations of *C. elegans* (San-Miguel and Lu, 2013; Midkiff and San-Miguel, 2019), and microfluidic devices capable of immobilizing *C. elegans* without the need for invasive, chemical paralysis have been most highly sought after. These include the on-chip use of temperature-sensitive gels (Krajniak and Lu, 2010), quake valves, and deformable membranes that collapse and compress the nematode (Chokshi et al., 2009; Keil et al., 2017), or suction channels that trap the nematodes near a side wall (Rohde et al., 2007; Gokce et al., 2014). However, many of these approaches, unfortunately, rely on additional specialized equipment, thus, reducing accessibility, or solely focus on the immobilization of individual specimens (Hu et al., 2013; Lockery et al., 2012). Hence, we decided to use passive immobilization. In contrast to previous approaches (Hulme et al., 2007; Mondal et al., 2016), our device is specifically designed for manual operation and builds on straight channels with constant widths followed by a constriction, rather than tapered channels, as *C. elegans* have been observed pushing themselves off angled side walls without the constant application of a vacuum. Further, given its simple application, our device even permits the release of the nematodes post imaging, thus, enabling investigations throughout their lifespan.

## MATERIALS AND METHODS

### Cloning of pPD95_77::rgef-1p::GCaMP6m and mScarlet-Tau-IRES constructs

The pan-neuronal expression of the GCaMP6m was achieved by amplifying the *GCaMP6m* sequence from the pN1-GCaMP6m-XC (Addgene #111543) plasmid with following primers: 5′-CGACGACGACGACGGCTAGCATGGGTTCTCATCATCATCATCATCAT-3′ and 5′-ACGGGCGCGAGATGCGGCCGCTCACTTCGCTGTCATCATTTGTACAAACTC-3′. The sequence of the pan-neuronal promotor *rgef-1* promoter was amplified of pPD95-DBN(wt)-YFP 17 with following primers: 5′-ACACTGCAGCATGCAAGACTAATTTTCG-3′ and 5′-ATAGGATCCGTCGTCGTCGTCGAT-3′ and cloned into the backbone of pPD95_77 (Addgene #1495) (pPD95_77::*rgef-1p*). The GCaMP6m PCR product and the pPD95_77::*rgef-1p* were digested with NheI and NotI and Gibson Assembly (NEB) was carried out to obtain pPD95_77::*rgef-1p::GCaMP6m*.

The construct for neuronal expression of Tau^WT^ with sub-stoichiometric expression of mScarlet::Tau^WT^ and of Tau^P301L,V337M^ mScarlet::Tau^P301L,V337M^ sub-stoichiometric expression of mScarlet::Tau^P301L,V337M^ were generated by assembling four amplified fragments through Gibson Assembly (NEB).

The human *Tau^WT^* gene was amplified from the pRK5-EGFP-Tau (a gift from Mark Hipp, UMC Groningen, The Netherlands) plasmid, with the primers 5′-GACGACCGGGATGGCTGAGCCCCGCCAGGAG-3′ with homologous overlaps to the *rgef-1* promotor sequence of the plasmid backbone at the 5′-end and 5′-AGCAACCGGTTCACAAACCCTGCTTGGCCAG-3′ with homologous overlaps to the *hsp-3^IRES^* sequence at the 3′-end. The *hsp-3^IRES^-mScarlet* sequence was amplified from the *pPD95_rgef-1p::SigPep-A*β*1−42-IRES-mScarlet-A*β*1−42::unc-54(3′UTR)* (Gallrein et al., 2021) with primers 5′-GGGTTTGTGAACCGGTTGCTCTCCCTCAC-3′ with homologous overlaps to the *Tau^WT^* sequence at the 5′-end and 5′-GCTCAGCGGAGCTAGCCTTGTAGAGCTCGTC-3′ with homologous overlaps to the *Tau^WT^* sequence at the 3′-end. The human *Tau^WT^* gene for the expression of the Tau^WT^ sub-stoichiometrically tagged to the mScarlet fluorophore was amplified from the pRK5-EGFP-Tau plasmid with the primers 5′-CAAGGCTAGCTCCGCTGAGCCCCGCCAG-3′ with homologous overlaps to the *hsp-3^IRES^-mScarlet* sequence at the 3′-end and 5′-GTATCTCGAGTCACAAACCCTGCTTGGCCAGG-3′ with homologous overlaps to the plasmid backbone at the 3′-end. The plasmid backbone containing the *rgef-1* promotor sequence was amplified from plasmid *pPD95_rgef-1p::SigPep-A*β*1−42-IRES-mScarlet-A*β*1−42::unc-54(3′UTR)* (Gallrein et al., 2021) with the primers 5′-GGGTTTGTGACTCGAGATACCCAGATCATATGAAACGGC-3′ with homologous overlaps to the *Tau^WT^ sequence* at the 5′-end and 5′-GCTCAGCCATCCCGGTCGTCGTCGTCGT-3′ with homologous overlaps to the *Tau^WT^* sequence at the 3′-end.

The human *Tau^P301L,^*^V337M^ gene was amplified from the pN1 FLTau0N4RLM YFP (a gift from Mark Hipp) plasmid with the primers 5′-GACGACCGGGATGGCTGAGCCCCGCCAG-3′ with homologous overlaps to the *rgef-1* promotor sequence of the plasmid backbone at the 5′-end and 5′-AACCGGTTTACAAACCCTGCTTGGCCAGG-3′ with homologous overlaps to the *hsp-3^IRES^* sequence at the 3′-end. The *hsp-3^IRES^-mScarlet* sequence was amplified from the *pPD95_rgef-1p::SigPep-A*β*1−42-IRES-mScarlet-A*β*1−42::unc-54(3′UTR)* (Gallrein et al., 2021) with the primers 5′-GCAGGGTTTGTAAACCGGTTGCTCTCCC-3′ with homologous overlaps to the *Tau^P301L,^*^V337M^ sequence at the 5′-end and 5′-GCTCAGCGGAGCTAGCCTTGTAGAGCTC-3′ with homologous overlaps to the *Tau^P301L,^*^V337M^ sequence at the 3′-end. The human *Tau^P301L,V337M^* gene for the expression of the Tau^P301L,V337M^ sub-stoichiometrically tagged to the mScarlet fluorophore was amplified from the pN1 FLTau0N4RLM YFP plasmid with the primers 5′-CAAGGCTAGCTCCGCTGAGCCCCGCCAGGAG-3′ with homologous overlaps to the *hsp-3^IRES^-mScarlet* sequence at the 3′-end and 5′-TCTCGAGCTACAAACCCTGCTTGGCCAGGG-3′ with homologous overlaps to the plasmid backbone at the 3′-end. The plasmid backbone containing the *rgef-1* promotor sequence was amplified from the plasmid *pPD95_rgef-1p::SigPep-A*β*1−42-IRES-mScarlet-A*β*1−42::unc-54(3′UTR)* (Gallrein et al., 2021) with the primers 5′-GCTCAGCCATCCCGGTCGTCGTCGTCGT-3′ with homologous overlaps to the *Tau^P301L,V337M^* sequence at the 5′-end and 5′-GCTCAGCCATCCCGGTCGTCGTCGTCGT-3′ with homologous overlaps to the *Tau^P301L,V337M^* sequence at the 3′-end.

Gibson Assembly was performed following the manufacturer's instructions to create the final plasmids that contain all 4 fragments. (*pPD95_rgef-1p::Tau^WT^-IRES-wrmScarlet-Tau^WT^::unc-54(3′UTR)* and *pPD95_rgef-1p::Tau^P301L,V337M^-IRES-wrmScarlet-Tau^P301L,V337M^::unc-54(3′UTR)*).

All cloned constructs were confirmed by sequencing (LGC Genomics, Berlin, Germany).

### *C. elegans* strains

All *C. elegans* strains generated or used for this work are listed in Table S1.

### Generation of new *C. elegans* strains

The newly generated transgenic strains nGCaMP6m, nTau^WT^ and nTau^P301L,V337M^ have been generated by micro particle bombardment using 10 μg of the respective plasmids into N2 wild-type nematodes. Established protocols were used (Gallrein et al., 2021; Wilm et al., 1999; Radman et al., 2013). Progeny was scored for red fluorescence, and positive nematodes were singled out. The extra-chromosomal arrays have been integrated into the genome by UV integration. For that, a full 10-cm plate of synchronized L4 stage nematodes carrying the extrachromosomal array were washed off nematode growth medium (NGM) plates with M9 buffer and transferred to unseeded NGM plates. Animals were exposed to UV irradiation using a dose of 0.012 J/cm^2^. Following irradiation, the nematodes were allowed to recover for 24 h at 20°C before being chunked to separate NGM plates. The F2 generation was then screened for positive transformants and stable inheritance of the transgene over multiple generations by singling out individual nematodes. The integrated strains were backcrossed six times with N2 wild-type nematodes.

### Genetic crosses

Male nematodes used for genetic crosses were generated by exposing L4 larvae to heat treatment (31°C for 7 h). Generated males were isolated from their offspring and maintained by continuous crossing with L4 hermaphrodites. Crosses were performed by transferring L4 hermaphrodites of one genotype with an excess of males (10:1 ratio) of the other genotype on an NGM-agar plate. The desired offspring was then isolated and checked for homozygous expression of the transgenes. The offspring of the cross of the nGCaMP6m strain and the MT8004 strain was validated by genotyping with primers 5′-TGACCACTTTGGAACCCCAT-3′ and 5′-GCATCGGAGTTTCAGTATTCTGTT-3′ by using the Phire Tissue Direct PCR Master Mix kit (Thermo Fisher). The amplicons were sequenced to check for homozygosity of the single point mutation. The offspring of the strain nTauWT and RB809 was validated by genotyping using the primers 5′-CCTCCTACCACCCATCTGAA-3′ and 5′-CAACATGCTCAGGGAAGTCA-3′.

### *C. elegans* maintenance

*C. elegans* nematodes were maintained at 20°C in the dark on solid nematode growth medium (NGM) seeded with live OP50 *E. coli*. All assays were performed at 20°C with age-synchronized nematodes. Young adult animals were referred to as day 4 of life. Animals were synchronized by egg-laying or by picking L4 larvae.

### RNA extraction, reverse transcription and real-time quantitative PCR

Nematodes were synchronized by picking 200 L4 stage nematodes of strains N2 (wild type), JKM150 (Tau^WT^), and JKM151 (Tau^P301L,V337M^). Age synchronized nematodes were grown for one, four or seven days, rinsed off from the culture plates with M9, washed three times with M9, resuspended in 1 ml Trizol and snap frozen in liquid nitrogen. Frozen nematode samples were thawed and lysed during three cycles of 6000 rpm shaking for 3 s in a Precellys homogenizer. To this, 100 µl bromo-chlor-propane was added, vortexed for 15 s and centrifuged at 12,000 ***g*** for 15 min at 4°C. The clear phase was used for RNA extraction with the RNeasy Mini Kit (QIAGEN). RNA concentration was measured with Nanodrop and RNA quality was assessed using a 1% agarose TAE gel. The SuperScript^®^ III Reverse Transcriptase (Invitrogen) kit was used for reverse transcription reactions. 1 μg of RNA was diluted in 11.5 μl total volume with RNase-free H_2_O. The mixture was heated to 70°C for 2 min and kept on ice afterwards. 8.5 μl master mix [4 μl 5× Buffer, 2 μl DTT, 1 μl dNTP, 1 μl Oligo dT, and 0.5 μl SS III RT enzyme (Invitrogen)] was added to the RNA. The sample was incubated at 25°C for 5 min, followed by 60 min incubation at 50°C, and heat-inactivation at 95°C for 15 min. 80 μl of ddH_2_O was added to the cDNA sample. For measuring Tau expression 10 μl PowerUp™ SYBR™ Green Master Mix was mixed with 10 mM forward and reverse primers (see below), 2 μl cDNA per sample was added and the reaction filled up with ddH_2_O to 20 μl. Samples were measured in triplicates. Four cohorts have been analyzed. Quantitative PCR (qPCR) was performed by using CFX96 Touch real-time PCR detection system (BioRad). Target genes expression was normalized to expression of the reference genes (*act-1*, *lmn-1*, *eif-3.C*). The data were obtained by CFX Manager Software (BioRad) and processed with Excel 2016 (Microsoft). Primer sequences were: qPCR_act-1--for 5′-CCACCATGTACCCAGGAATT-3′; qPCR_act-1--rev 5′-AGAGGGAAGCGAGGATAGAT-3′; qPCR_lmn-1--for 5′-CATCTCGTAAAGGTACTCGTAG-3′; qPCR_lmn-1--rev 5′-GTTGAGCCAAATGAATCGTC-3′; qPCR_eif-3.C--for 5′-ACACTTGACGAGCCCACCGAC-3′; qPCR_eif-3.C--rev 5′-TGCCGCTCGTTCCTTCCTGG-3′; qPCR_hTau-mScarlet-pair07--for 5′-ACCGTAAGCTCGACATCACC-3′; qPCR_hTau-mScarlet-pair07--rev 5′-GTCCCAGCGTGATCTTCCAT-3′.

### Protein extraction from nematodes

Nematodes were lysed in boiling in Laemmli loading buffer. 100-125 animals of the respective age were picked into 1 ml of M9 buffer and washed three times in a low-binding tube (Sarstedt). Lysis was performed in 20 μl M9 mixed with 30 μl 4× Laemmli loading buffer at 99°C and while shaking for 10 min at 1000 rpm.

### Western blotting of nematode lysates to quantify GCaMP6m protein levels

Nematode lysate was separated using 10% SDS-PAGE and transferred onto a PVDF membrane using the Trans-Blot Turbo Transfer System (Bio-Rad) and the standard program for 30 min at 25 V. Antibodies used were anti-GAPDH (Proteintech, 1:20,000), anti-GFP(B34)/GCaMP6m (Enzo, 1:1000) and anti-Tau (cat. no.: MA5-12808, Thermo Fisher, 1:200). Proteins were detected via chemiluminescence using ECL-reagent and the ChemoStar device (Intas). Signal intensities were quantified by Fiji and normalized to GAPDH. Data are displayed as relative values to a control sample (i.e. nmScarlet, mmScarlet, nGCaMP6, nGCaMP6m DMSO treatment).

### Lifespan assay

100-180 L4 larvae were transferred in cohorts of 10-15 animals onto seeded NGM agar plates. Lifespan assay was performed as described previously (Gallrein et al., 2021). In brief, animals were transferred to fresh plates and scored daily for alive, dead or censored animals until all animals died. Animals that escaped the plates or showed a bagging phenotype were censored. Nematodes were transferred to fresh plates until day 9 to separate them from their offspring. Survival was calculated with the online tool OASIS 2 (Han et al., 2016). Experiments were carried out in triplicates.

### Progeny assay

For each strain, 30-40 L4 larvae were isolated onto seeded 35 mm NGM agar plates and transferred daily onto fresh plates until they stopped laying eggs. For every nematode, the number of viable offspring was scored. Experiments were carried out in triplicates.

### Chemotaxis assay

Nematodes were synchronized and grown until the desired age and washed from the plates with M9 buffer at least five times to remove any residual bacteria. Nematodes were kept in M9 followed by a starvation period of 90 min at 20°C in the dark. In the meantime, unseeded NGM agar plates were marked with four quadrants and a circle in the center. Two opposite quadrants were marked as tests and 2 µl of odorant sample substance was added. The other two quadrants were marked as controls and 2 μl of control substance was added. Odorant sample substances were mixed with sodium azide (NaN_3_, 500 mM) in a 1:1 (v/v) ratio with either pure benzaldehyde (test repellent) or with diluted benzaldehyde (2% v/v in water) (test attractant). H_2_O was used as control substance as a mix with NaN_3_. Of the pelleted nematodes 5-10 μl were pipetted onto the center of the prepared plates to test around 80-100 nematodes. The plate was incubated in the dark at 20°C for 2 h. Afterwards, nematodes were counted in each quadrant, and the chemotaxis index was calculated as the difference between the fraction of nematodes on the sample quadrants and on control quadrants, divided by the total number of nematodes counted. Experiments were carried out in three to four biological replicates, and for each cohort three attraction and repulsion plates were used.

### Developmental assay

For each strain, 60 eggs were placed onto seeded 35-mm NGM agar plates. Nematodes that did not hatch were excluded from the analysis. The start of the experiment was considered as day 1. The developmental stage of each nematode was checked daily until adulthood was reached. A nematode was considered adult when the first fertilized eggs were observed in the gonads. Experiments were performed in triplicates.

### Thrashing assay

Nematodes were analyzed in M9 medium on day 4 and 10 of life. Nematodes were synchronized and grown to the desired age. 3 ml of M9 medium was added to an empty 35-mm plate and 10-15 nematodes were picked into the liquid. The nematodes were allowed to swim for 3 min before video recording was started. Videos were recorded at 7.3× magnification at a frame rate of 30 frames per second using a stereomicroscope equipped with a camera and recording software (Leica M165 FC). Each group of nematodes was recorded three times for 20 s with an exposure time of 1 ms. Videos were analyzed using the WormLab software (MBF Bioscience), using the bending angle (midpoint) analysis option with an amplitude threshold of 20° and a duration threshold of 10 s. A nematode was only included in the final analysis if the tracking lasted for at least 50% of the frames. Frequency values provided by the WormLab software were exported to Excel.

### Neurodegeneration assay

For quantification of neurodegeneration, adult nematodes of day 7 of life of the strains JKM188 (nmScarlet), JKM187 (Tau^P301L,V337M^), JKM220 (TauWT) were analyzed. Animals were mounted on microscope slides using 3% agarose and anesthetized with 250 mM NaN_3_. Z-stacks of PLML and PLMR neurons were acquired using a Zeiss LSM710 Confocor3 confocal microscope. Neurodegeneration was assessed manually using ZEN Blue software (version 3.8.99.01000, Zeiss). The following morphological abnormalities were used as criteria for neurodegeneration: blebs, axonal and somatic outgrowths, interruptions (necrosis) and wavy structures. Each animal was categorized into one of two degeneration groups: no degeneration/ slight degeneration or strong degeneration. Thresholds for strong degeneration were defined as the presence of at least one of the following: ≥12 blebs, ≥2 axonal outgrowths, ≥1 somal outgrowth, ≥1 interruption (necrosis) or ≥1 wavy structure. Animals exhibiting strong degeneration were used for statistical analysis between the strains using Fisher's exact test.

### Design and fabrication of the microfluidic mold

The design of the microfluidic channel consisted of 16 parallel microchannels, which were connected to a shared inlet and outlet via regular branching (Fig. S1). Following this approach, all channels were of the same length with equal pressure loss, hence, promoting the separation of the individual nematodes after their introduction into the device. Additionally, the large number of channels permitted the distribution of the volume flow as well as the reduction of on-chip fluid velocities and, by that, allowed for a higher controllability during the loading and trapping procedure without the need for microfluidic pumps. Depending on the age of the investigated nematodes, the widths of the parallel microchannels differed between 40 μm (day 4) and 60 μm (days 7 and 10), while the height of all channels was 65 μm. Additionally, to prevent the nematodes from getting flushed through the device during loading, each microfluidic channel was interrupted by a constriction by which its width was reduced to 12 or 20 μm, respectively. Further dimensions of the design can be found in Fig. S1A; the corresponding lithography mask was designed in CleWin 4.0 Layout Editor (WieWeb Software) and printed on film (JD Photo Data). It is worth noting that, while tested, no pillars were introduced near the entrance of the final design as we were unable to confirm their previously described advantages on *C. elegans* orientation (Hu et al., 2013).

The microfluidic mold was fabricated by using single-layer photolithography as described previously (Fig. S1C) (Läubli et al., 2021). First, a single-side polished silicon wafer was cleaned using acetone, isopropanol and de-ionized H_2_O, before being pre-baked at 200°C for 10 min. Once cooled to room temperature, SU-8 50 negative photoresist (Kayaku Advanced Materials) was manually dispensed onto the wafer for spin coating. The photoresist was first distributed at 500 rpm for 15 s with an acceleration of 200 rpm/s before being reduced to the final thickness by using 1800 rpm for 30 s with an acceleration of 300 rpm/s. The wafer was then transferred to a hotplate for soft baking at 65°C for 7 min before the temperature was ramped to 95°C for a further 22 min. Finally, the wafer was cooled down to 75°C on the hotplate before being removed and left to cool to room temperature for further processing. To transfer the design, the photoresist was exposed to 365 nm UV under contact mode for 40 s using an EVG620 NT mask aligner until a total exposure dose of 400 mJ/cm^2^ was reached. The resist was post-exposure baked at 65°C for 1 min before being moved to another hotplate for 6 min at 95°C and cooled down to room temperature. The design was developed in a bath with SU-8 Developer (Kayaku Advanced Materials) for 5 min, using manual agitation before development was stopped by rinsing the wafer with isopropanol followed by de-ionized H_2_O and dried using a nitrogen gun. To reduce stress inside the material, the resist was baked hard at 150°C for 10 min before being cooled down to room temperature. Finally, the wafer was coated with Tridecafluoro-1,1,2,2-tetrahydrooctyl)trichlorosilane (CAS 78560-45-9, abcr) under vacuum for 1 h to increase reusability.

### PDMS microchannel fabrication for *C. elegans* imaging

Preparation of polydimethylsiloxane (PDMS) was carried out by combining Sylgard 184 curing agent and Sylgard 184 base elastomer (Dow) at a ratio of 1:10 (w/w) followed by vigorous mixing for 5 min. The mixture was poured over the SU-8 mold (Fig. S1C) and the PDMS was degassed in a desiccator for ∼ 30 min until no air bubbles were visible. The PDMS was solidified in an oven at 50°C overnight, before being cut and carefully peeled off the wafer. The wafer-sized PDMS was then separated into single devices using a razor blade. In- and outlets of 1.5 mm were applied using a biopsy punch (Ted Pella). Dust was removed from individual devices using Scotch tape before they were cleaned using soap water (Decon 90, Decon Laboratories), 70% ethanol and ultrapure H_2_O. To remove any residual water or ethanol, PDMS devices were dried using compressed air and incubated on a hot plate at 120°C for 5 min. Bonding of PDMS devices to glass coverslips (No. 1.5) was performed using a plasma asher (Zepto, Diener Electronic). For that, the surfaces of the glass and the PDMS were exposed to the air plasma for 30 s before being brought into contact immediately under gentle pressure. The bond was then stabilized by baking the devices at 80°C for 5 min on a hotplate.

To load the nematodes into the PDMS channels a 1 ml low-binding pipette tip was placed at the inlet and a syringe with a flexible tubing was added at the outlet. The syringe was filled with M9 media, and the channels flushed from the outlet until the pipette at the inlet was filled half. Nematodes were picked from plates into the M9 reservoir within the pipette tip and worms were allowed to sink by gravity. By pulling slightly on the syringe, the nematodes entered the device and were separated into the microchannels where they were trapped along the sides of their bodies.

### Imaging of GCaMP6m intensity and normalization of intensities to protein levels

GCaMP6m intensities of nematodes were recorded on days 4, 7 and 10 of life, with day 1 being the day of synchronization. Nematodes were synchronized and grown until the desired age and loaded onto the fabricated PDMS microfluidic devices as described above in groups of up to six animals. For 4-day-old nematodes, channels with a width of 40 μm were used, while older animals (day 7 and 10) were imaged in 60-μm-wide channels. Images of individual nematodes were taken using a custom-built widefield microscope (IX83, Olympus) with an sCMOS camera (Zyla 5.5, Andor) and a 10× objective (Plan N 10×/0.25, Olympus). GCaMP6m was excited at 470 nm from a four-wavelength high-power light emitting diode light source (LED4D067, Thorlabs). To record GCaMP6m of individual nematodes, two sets of image series comprising ten images each were taken with an exposure time of 170 ms using the software Micro-Manager (Edelstein et al., 2014). Additionally, a brightfield image series of each nematode was taken as a reference with an exposure time of 10 ms. Fluorescence intensities of single nematodes were quantified using Fiji by selecting the most focused and stable image out of the two fluorescence image series. Normalization of the acquired GCaMP6m intensities to the GCaMP6m protein levels was performed to account for the varying expression of GCaMP6m in different strains. Based on the GAPDH-normalized GCaMP6m protein levels, a normalization factor was calculated by dividing the mean expression level of control nematodes (i.e. neuronal- or muscular-expressing mScarlet strains) by the value for Ab/Tau-expressing nematodes on days 4, 7 and 10 of life. Subsequently, the GCaMP6m intensity values were multiplied by the normalization factor, resulting in GCaMP6m intensities normalized to protein levels.

### Nemadipine A treatment

The L-type Ca^2+^-channel inhibitor nemadipine A was dissolved in DMSO. Nemadipine A or DMSO as a solvent control was added to the liquid NGM agar at a final concentration of 2 µM before pouring. Nematodes were synchronized by egg-laying on agar plates containing nemadipine A or DMSO and grown to the desired age in the presence of nemadipine A or DMSO. nGCaMP6m intensities of nemadipine A- and DMSO-treated animals were recorded using an LSM-880 (Zeiss) confocal laser scanning microscope. Animals were immobilized on a glass slide containing a 10% agarose pad using 0.1 µm polystyrene microbeads (Polyscience 00876-15). Analysis and normalization were performed as described above.

### Confocal fluorescence imaging

Animals of a defined age were anesthetized with 250 mM NaN_3_ and mounted on a glass slide with 3% (w/v) agarose pads. Confocal images were obtained using a laser-scanning microscope LSM-880 (Zeiss). Whole nematode images were acquired using an EC Plan-Neofluar 10×/0.3 M27 objective, while head, tail, mid body and coelomocytes images were obtained using a Plan-Apochromat 40×/1,4 Oil DIC M27 objective. GFP was excited at 488 nm using an Argon laser, while mScarlet was excited at 543 nm using the HeNe laser. Transmitted-light images of the nematodes (T-PMT Transmission) were overlayed with the fluorescence image. Fluorescence intensities were quantified using Fiji by selecting nematodes individually and calculated by subtracting the product of the area selected and the average fluorescence of 3 background readings from the integrated density. Data are displayed as relative values to as control sample (i.e. nTau^WT^).

### Fluorescence lifetime imaging microscopy (FLIM)

Nematodes expressing nmScarlet or mScarlet-nTau^WT^/mScarlet-nTau^P301L,V337M^ of a defined age (day 4 and 10) were anesthetized with 250 mM NaN_3_ and mounted on an 3% agarose pad. Fluorescence lifetime imaging microscopy (FLIM) was performed using a confocal laser scanning microscope LSM-880 (Zeiss) with a pulsed laser (PicoQuant). The fluorescence lifetime was measured by time correlated single photon counting (TCSPC). mScarlet was excited with a 560 nm pulsed laser at 40 MHz with 60% laser intensity. Emission was recorded in the 575-625 nm range. Lifetime was monitored in the head neurons with a Plan-Apochromat 40×/1,4 Oil DIC M27 objective. Measurements were performed until the photon count reached 3000 photons in the brightest pixel by using SymPhoTime 64 Software. FLIM data were analyzed using FlimFit (5.1.1) software with pixel-wise fitting and assuming a mono-exponential decay. FLIM images were saved while lifetime values (τ) and histograms were exported to Excel.

### Statistical analysis

GraphPad Prism version 9 was employed for statistical analysis: Student's *t*-test with Welch's correction and one-way ANOVA with Bonferroni *post hoc* test were performed as parametric tests. Two-way ANOVA was performed to test significance according to two independent variables. As non-parametric tests, Mann−Whitey test, and Kruskal–Wallis test with Dunn's test were performed. Normality was tested by D'Agostino Pearson normality test. Significance for lifespan analysis was tested with a log-rank test using Oasis 2 online tool (Han et al., 2016). *P*-values: ns, *P*>0.05; *P≤0.05; **P≤0.01; ***P≤0.001; ****P≤0.0001.

## Supplementary Material

10.1242/dmm.052295_sup1 Supplementary information
