# Supplementary material for: Impairment of neuronal activity occurs at the early stages of the aggregation cascade of Aβ1-42 and mutant Tau
Source: Dis Model Mech. 2026 Apr 1;19(3):dmm052295. doi: 10.1242/dmm.052295 (PMC13072132; doi:10.1242/dmm.052295)
Supplement: Supplementary information [file dmm-19-052295-s1.pdf]

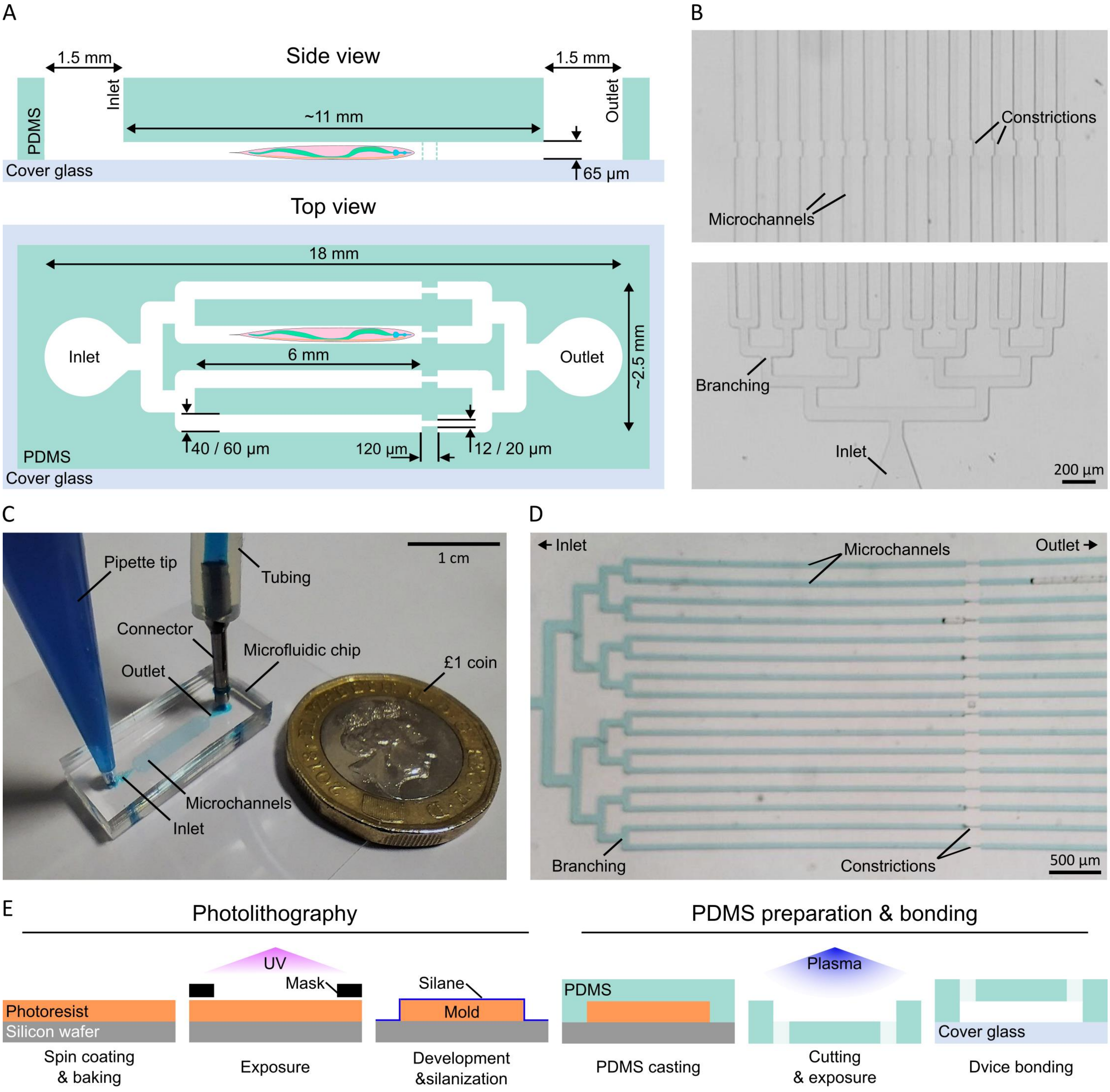

Fig. S1. Microfluidic design and fabrication.

A. Side and top view schematic of the microfluidic device. The PDMS-based device consists of 16 parallel microchannels with, depending on the age of the immobilized nematodes, widths of either 40 or 60 µm, while a uniform height of 65 µm is used throughout the device. The channels are connected to a shared inlet and outlet through regular branching. To prevent nematodes from getting flushed through the device, the channels are interrupted by constrictions.

B. Brightfield microscopy images of a microfluidic device with the inlet, branching, microchannels used for trapping as well as the constrictions labelled separately.

C. A photograph of the microfluidic chip next to a £1 coin for size reference. In line with the experimental procedure, a pipette tip as well as tubing have been added to the inlet and outlet, respectively. The device has been filled with blue food coloring for visualization purposes.

D. A micrograph of the same device with the 16 parallel microchannels being highlighted through food coloring. The complete area from the inlet over the branching to the constrictions is shown, which coincides with the region occupied by the *C. elegans* nematodes during the experiments.

E. The fabrication procedure for the microfluidic devices can be split in two main sections, with the first one being the photolithography leading to the fabrication of the mold and the second one being the PDMS casting, preparation, and bonding. First, SU-8 photoresist is spin coated onto a wafer and exposed to UV light to create the desired pattern. Following salinization of the mold, PDMS is casted and cured, before individual chips are prepared for bonding and, finally, sealed to glass cover slips through plasma treatment.

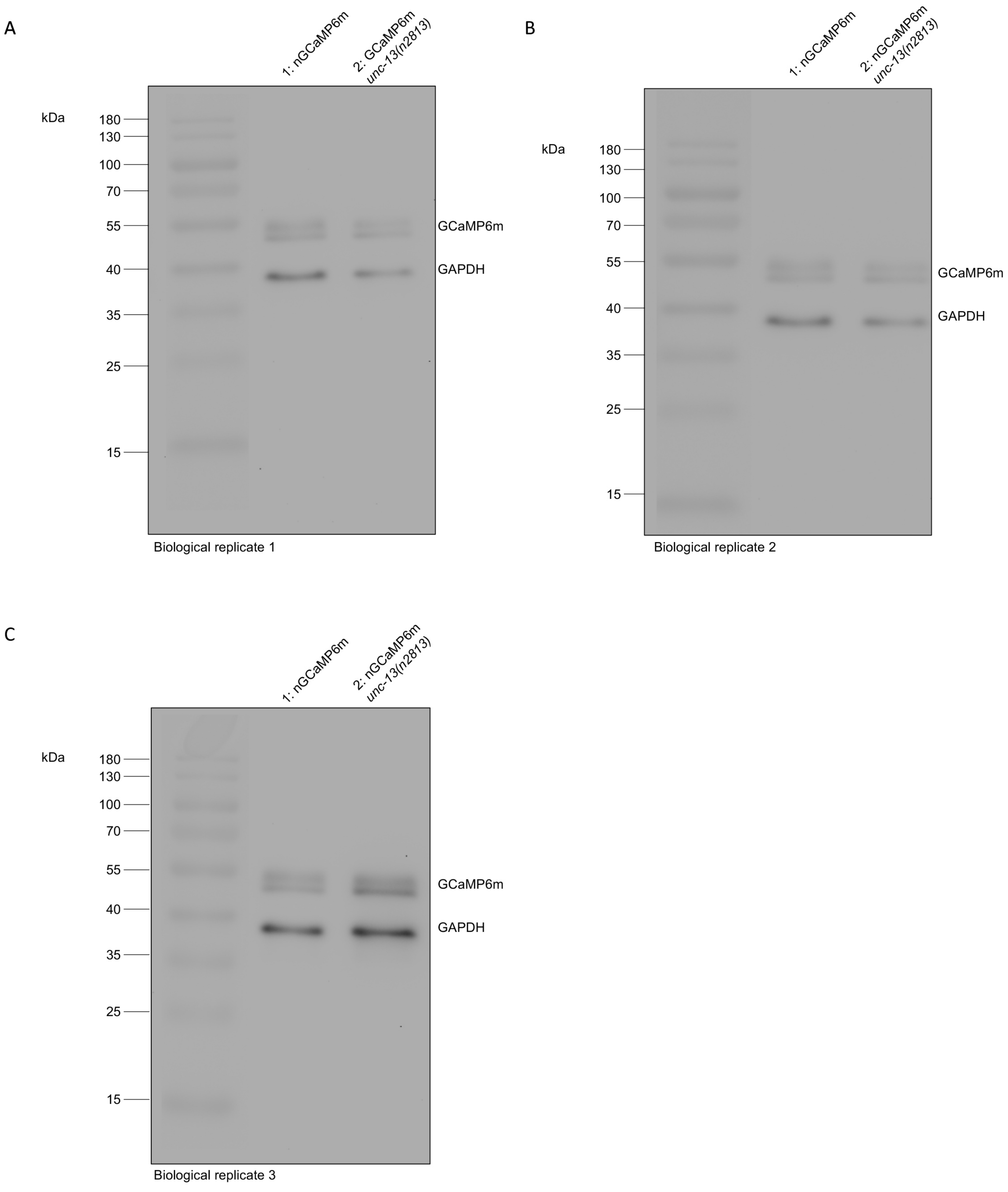

Fig. S2. Uncropped Western blots for the quantification of GCaMP6m protein levels in nGCaMP6m and nGCaMP6m *unc-13*(n2813) animals.

A. Western blot of the first biological replicate of crude protein lysates of nGCaMP6m (lane 1) and nGCaMP6m *unc-13(*n2813) (lane 2) animals. Protein bands of GCaMP6m and GAPDH and molecular weights of protein ladder (kDa) are labeled.

B. Western blot of the second biological replicate of crude protein lysates of nGCaMP6m (lane 1) and nGCaMP6m *unc-13*(n2813) (lane 2) animals. Protein bands of GCaMP6m and GAPDH and molecular weights of protein ladder (kDa) are labeled.

C. Western blot of the third biological replicate of crude protein lysates of nGCaMP6m (lane 1) and nGCaMP6m *unc-13*(n2813) (lane 2) animals. Protein bands of GCaMP6m and GAPDH and molecular weights of protein ladder (kDa) are labeled.

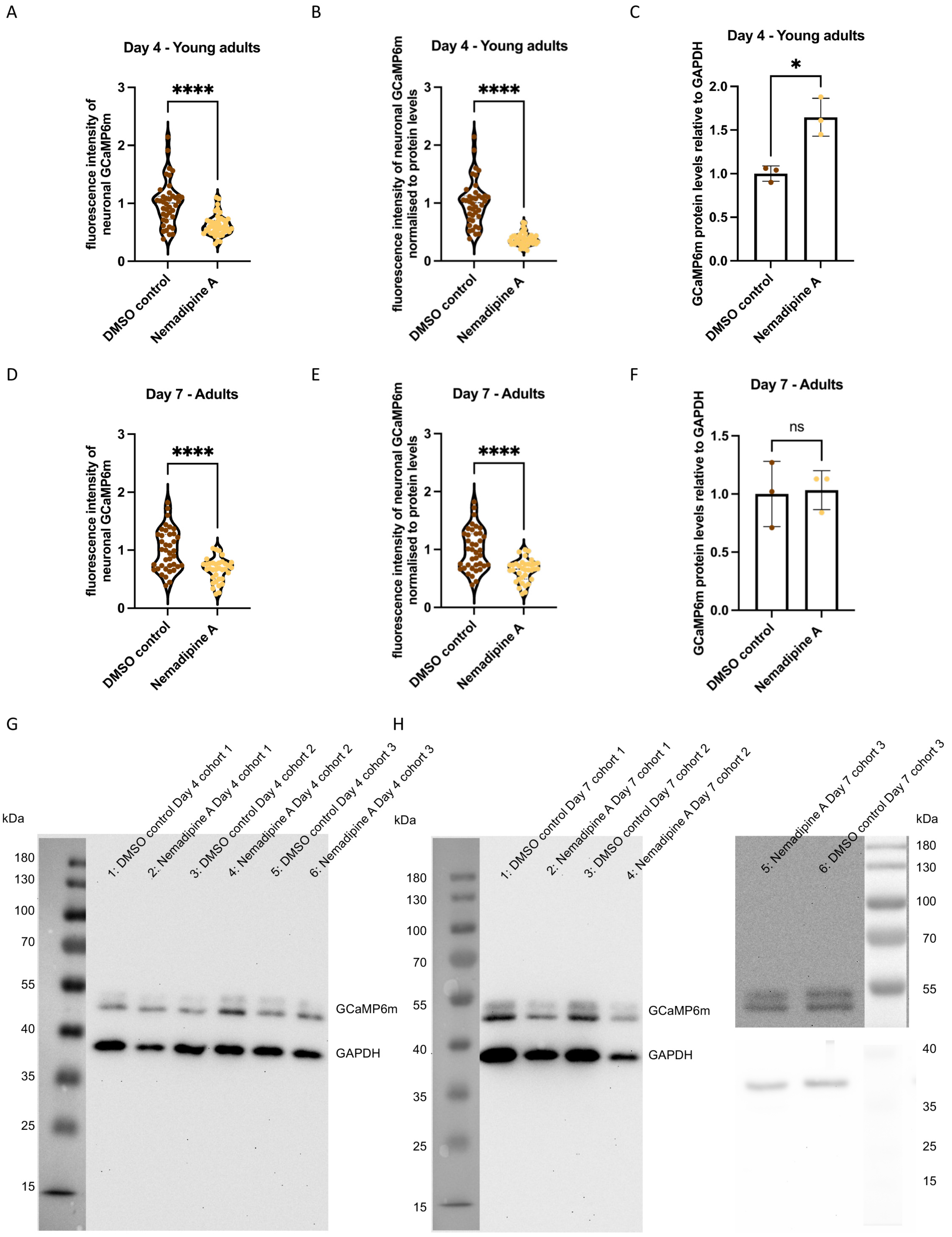

Disease Models & Mechanisms • Supplementary information

Fig. S3. The L-type calcium channel inhibitor Nemadipine A inhibits neuronal function.

A. Scatter dot plot of the average GCaMP6m fluorescence intensity of young adult animals (day 4-old) treated with Nemadipine A or with DMSO solvent control. GCaMP6m intensities were measured in alive animals immobilized with polystyrene beads with a confocal fluorescence microscope and intensities were quantified using Fiji. Every dot represents the neuronal GCaMP6m fluorescence intensity of a single animal treated with DMSO control (brown) and Nemadipine A (yellow). n = 3 and N = 40-46 animals. Significance was assessed by Mann-Whitney test (**** = $p \leq 0.0001$).

B. Scatter dot plot of the average GCaMP6m fluorescence intensity normalized to the GCaMP6m protein level of young adult animals (day 4 of life) treated with Nemadipine A or with DMSO solvent control. GCaMP6m intensities were measured in alive animals immobilized with polystyrene beads with a confocal fluorescence microscope and intensities were quantified using Fiji. GCaMP6m intensities were multiplied with the ratio between nGCaMP6m protein levels of the DMSO treated animals and Nemadipine A treated animals. Every dot represents the neuronal GCaMP6m fluorescence intensity normalized to GCaMP6m protein levels of a single animal treated with DMSO control (brown) and Nemadipine A (yellow). n = 3 and N = 40 - 46 animals. Significance was assessed by Mann-Whitney test (**** = $p \leq 0.0001$).

C. Quantification of GCaMP6m proteins levels by Western blot from total protein lysates of young adult (day 4 of life) treated with DMSO solvent control or Nemadipine A. Scatter dot plot shows quantification of GCaMP6m protein levels relative to GAPDH from three independent cohorts. Significance was assessed by unpaired Student's t-test with Welch's correction (* = $p < 0.05$).

D. Scatter dot plot of the average GCaMP6m fluorescence intensity of adult animals (day 7 of life) treated with Nemadipine A or with DMSO solvent control. GCaMP6m intensities were measured in alive animals immobilized with polystyrene beads with a confocal fluorescence microscope and intensities were quantified using Fiji. Every dot represents the neuronal GCaMP6m fluorescence intensity of a single animal treated with DMSO control (brown) and Nemadipine A (yellow). n = 3 and N = 35 - 39 animals. Significance was assessed by unpaired Student's t-test with Welch's correction (**** = $p \leq 0.0001$).

E. Scatter dot plot of the average GCaMP6m fluorescence intensity normalized to the GCaMP6m protein level of adult animals (day 7 of life) treated with Nemadipine A or with DMSO solvent control. GCaMP6m intensities were measured in alive animals immobilized with polystyrene beads with a confocal fluorescence microscope and intensities were quantified using Fiji. GCaMP6m intensities were multiplied with the ratio between nGCaMP6m protein levels of the DMSO treated animals and Nemadipine A treated animals. Every dot represents the neuronal GCaMP6m fluorescence intensity normalized to GCaMP6m protein levels of a single animal treated with DMSO control (brown) and Nemadipine A (yellow). n = 3 and N = 35 - 39 animals. Significance was assessed by unpaired Student's t-test with Welch's correction (**** = $p \leq 0.0001$).

F. Quantification of GCaMP6m proteins levels by Western blot from total protein lysates of adult animals (day 7 of life) treated with DMSO solvent control or Nemadipine A. Scatter dot plot shows quantification of GCaMP6m protein levels relative to GAPDH from three independent cohorts. Significance was assessed by unpaired Student's t-test with Welch's correction (ns = $p > 0.05$)

G. Western blot image of crude protein lysates from three biological replicates of young adult (day 4 of life) nGCaMP6m animas treated with DMSO control (lane 1, 3, 5) and Nemadipine A (lane 2, 4, 6) animals respectively. Protein bands of GCaMP6m and GAPDH and molecular weights of protein ladder (kDa) are labeled.

H. Western blot image of crude protein lysates from 3 biological replicates of adult (day 7 of life) nGCaMP6m animas treated with DMSO control (lane 1, 3, 6) and Nemadipine A (lane 2, 4, 5) animals respectively. Protein bands of GCaMP6m and GAPDH and molecular weights of protein ladder (kDa) are labeled. Samples of cohort 3 were analyzed on a separate gel, thus those protein bands are depicted separately from cohorts 1 and 2.

Disease Models & Mechanisms • Supplementary information

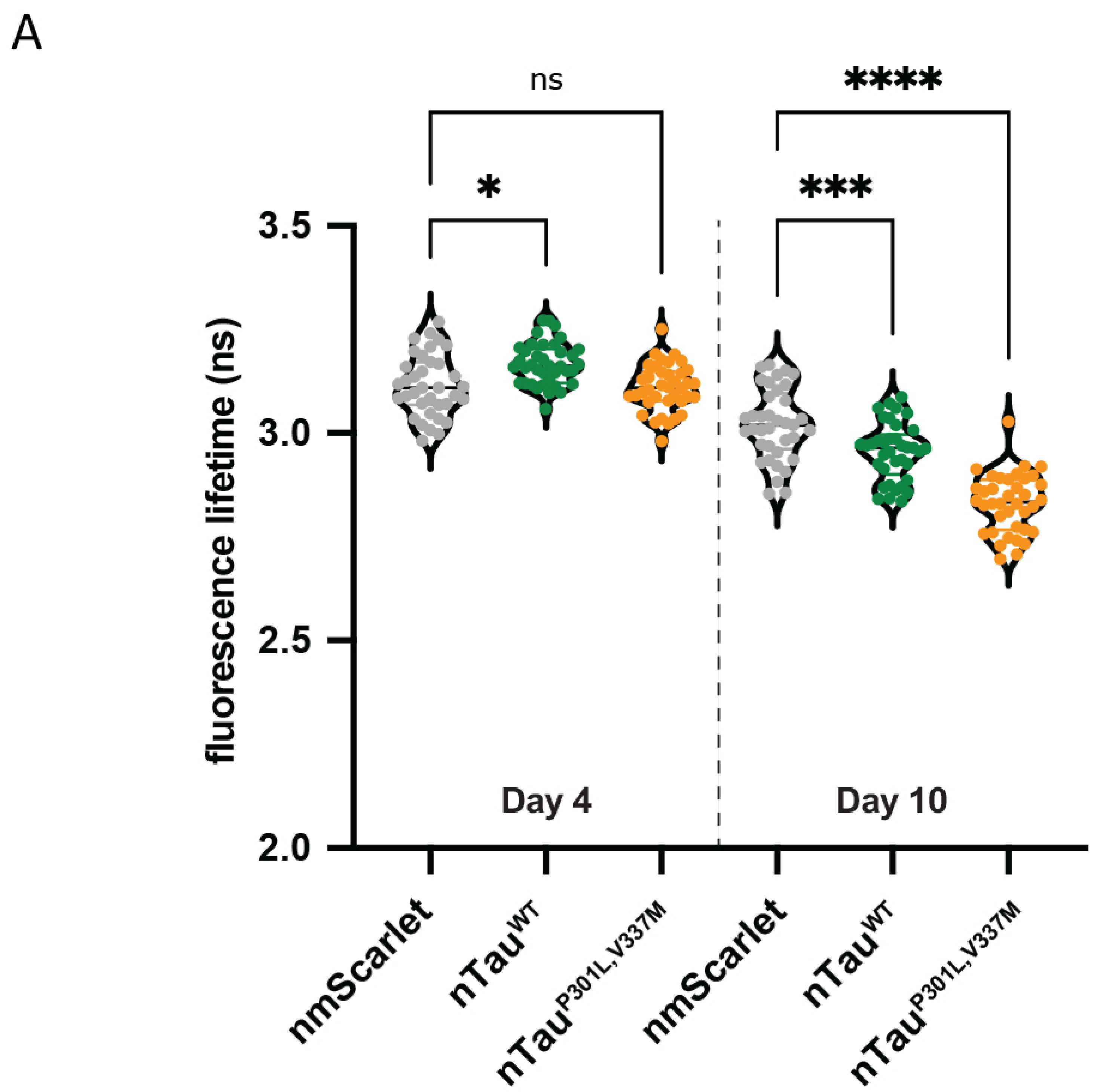

Fig. S4. Fluorescence lifetime analysis of neuronal Tau-expressing animals in the presence of neuronal GCaMP6.

Violine dot plot of the average fluorescent lifetime (τ) of young adult animals (day 4 of life; left) and old adult animals (day 10 of life; right) of nmScarlet, $nTau^{WT}$ and $nTau^{P301L,V337M}$ that also express GCaMP6m. Data displays average fluorescent lifetimes ± SD of nmScarlet (grey), $nTau^{WT}$ (green) and $nTau^{P301L, V337M}$ (orange). Every dot represents the average fluorescent lifetime for the head neurons of one single nematode. Three independent cohorts of in total 30 nematodes were analyzed. Significance was tested by one-way ANOVA + Bonferroni post hoc test for young adult animals and by Kruskal-Walls test with Dunn post hoc test for old animals (ns = $p > 0.05$; *** = $p \leq 0.001$; **** = $p \leq 0.0001$).

Disease Models & Mechanisms • Supplementary information

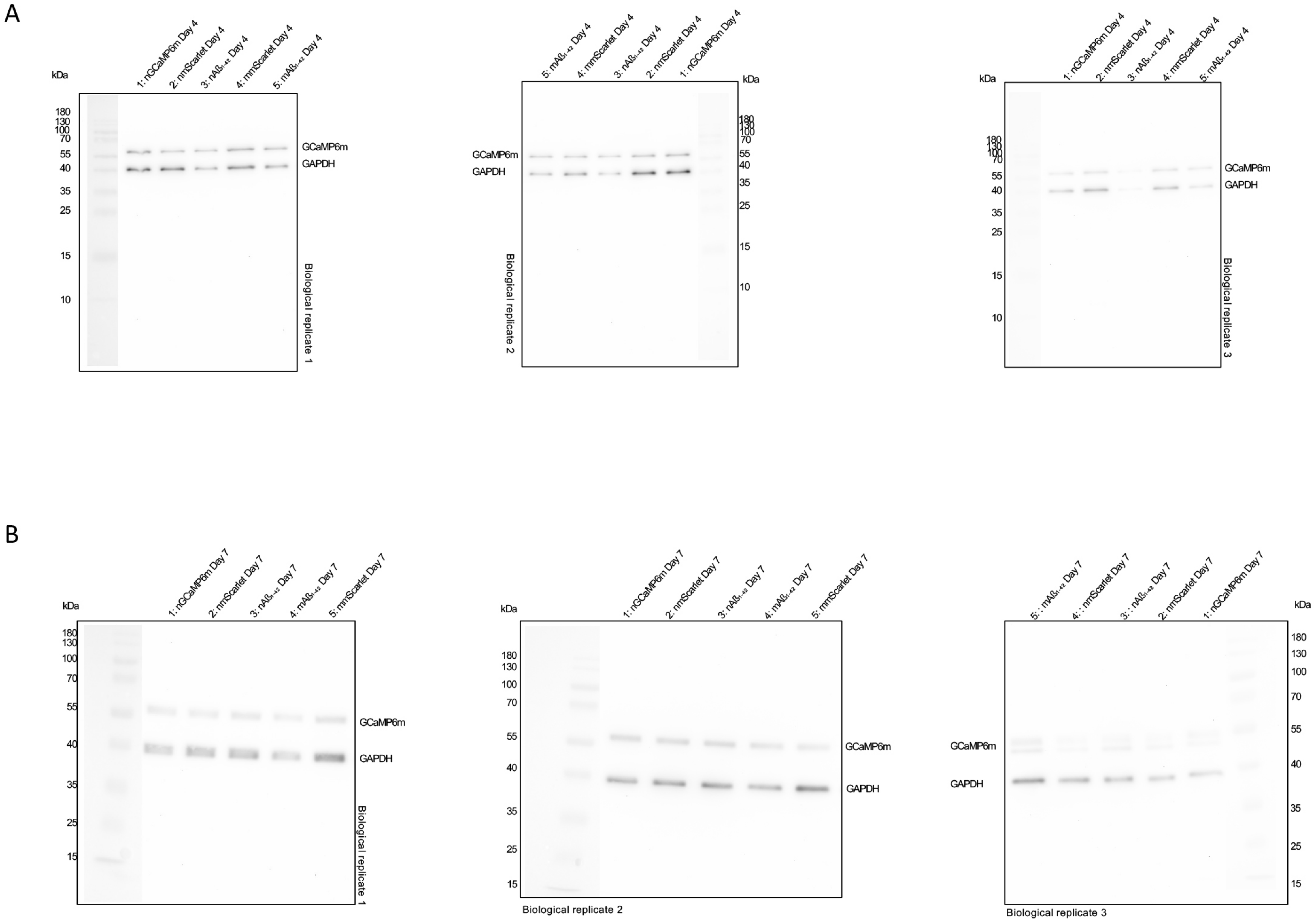

Fig. S5. Uncropped Western blots for the quantification of GCaMP6m protein levels in neuronal and muscle A$\beta_{1\text{-}42}$ and respective mScarlet control strains.
A. Western blots of the first, second and third biological replicate of crude protein lysates of young adult animals (day 4 of life) nGCaMP6m (lane 1), nmScarlet (lane 2), nA$\beta_{1\text{-}42}$ (line 3), mmScarlet (line 4) and mA$\beta_{1\text{-}42}$ (line 5) animals. Protein bands of GCaMP6m and GAPDH and molecular weights of protein ladder (kDa) are labeled.

B. Western blots of the first, second and third biological replicate of crude protein lysates of adult animals (day 7 of life) nGCaMP6m (lane 1), nmScarlet (lane 2), nA$\beta_{1\text{-}42}$ (line 3), mA$\beta_{1\text{-}42}$ (line 4) and mmScarlet (line 5) animals. Protein bands of GCaMP6m and GAPDH and molecular weights of protein ladder (kDa) are labeled.

Disease Models & Mechanisms • Supplementary information

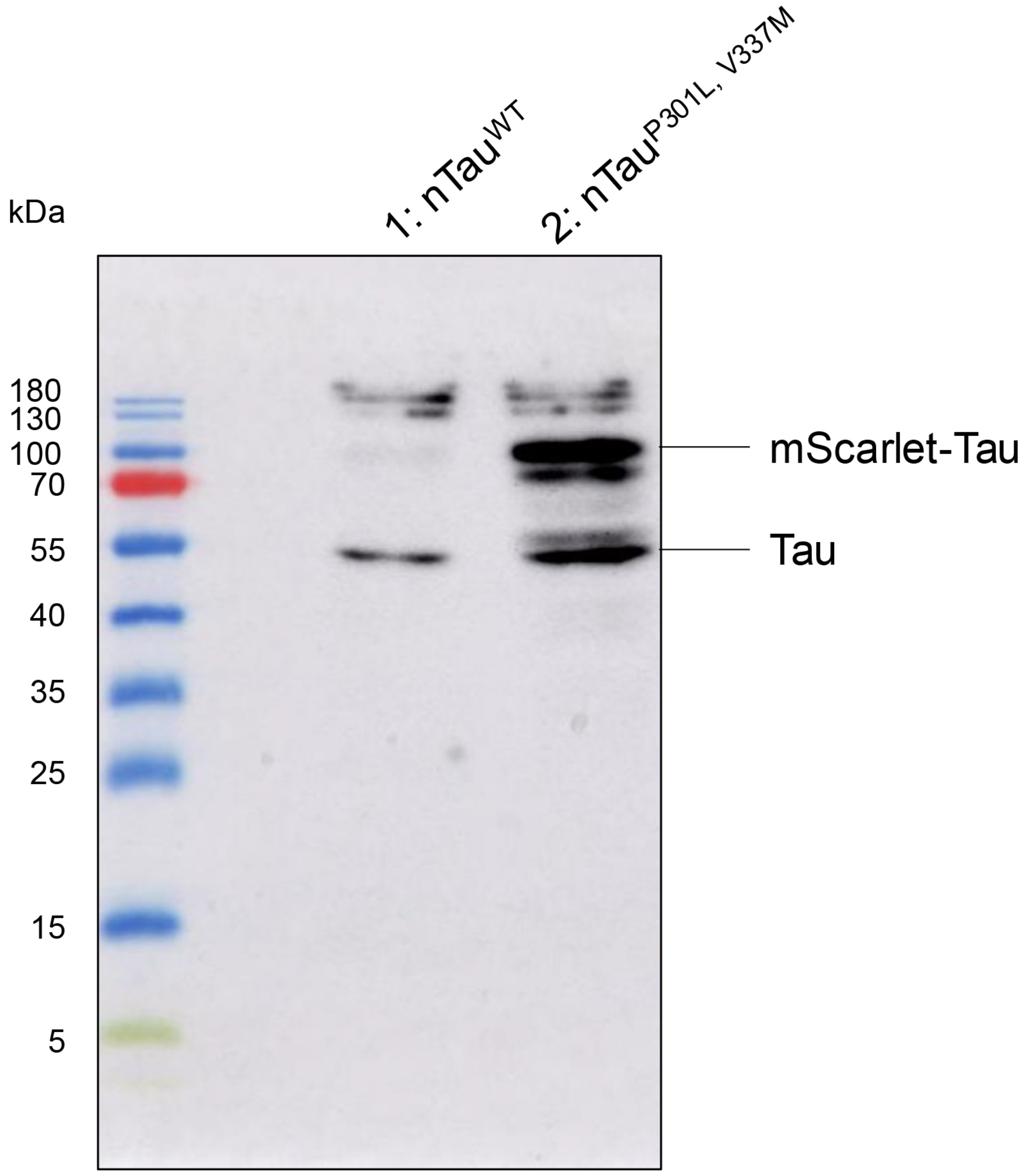

Fig. S6. Detection of untagged and mScarlet-tagged Tau protein from lysates of nTau^{WT} and nTau$^{P301L, V337M}$ animals.
Representative uncropped Western blot of protein lysates of nTau^{WT} (lane 1) and nTau^{P301L,V337M} (lane 2). Detection was performed with Mouse-anti-Tau-5 (MA5-12808, ThermoFisher) and Goat-anti-mouse-HRP. Protein bands of Tau, mScarlet-Tau and molecular weights of protein ladder (kDa) are labeled.

Disease Models & Mechanisms • Supplementary information

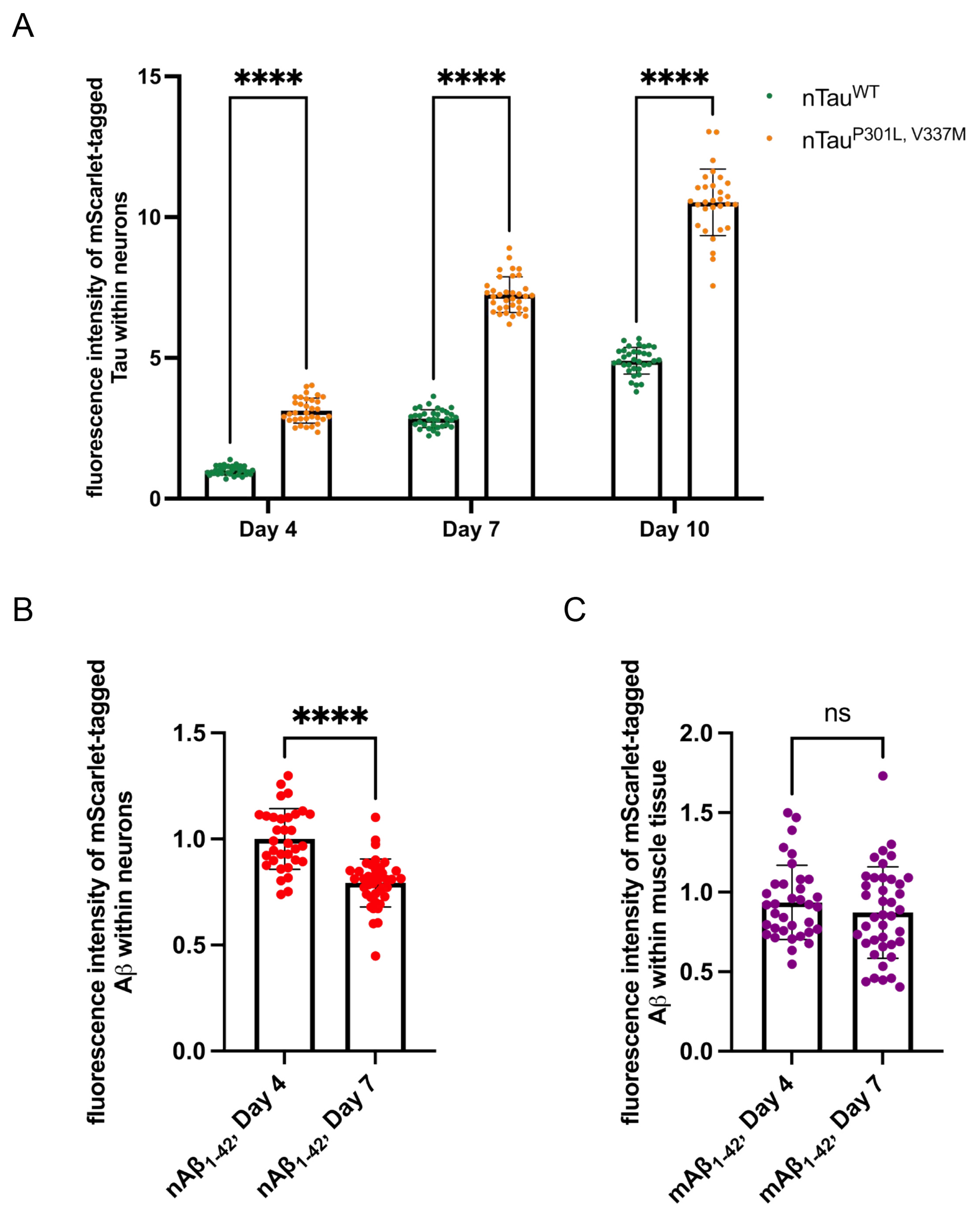

Fig. S7. Quantification of fluorescence intensity levels of mScarlet-tagged Tau / $A\beta_{1-42}$.

A. Quantification of mScarlet-Tau^{WT} and mScarlet-$Tau^{P301L,V337M}$ fluorescence intensity of young adult (day 4 of life), adult (day 7 of life) and old adult (day 10 of life) nematodes of the $nTau^{WT}$ and $nTau^{P301L,V337M}$ strain. Confocal fluorescent images of three cohorts of total 32 - 35 nematodes were recorded, and fluorescence intensities were quantified by Fiji. Data are displayed as mean fluorescence intensity ± SD. Significance was tested by two-way ANOVA + Bonferroni post hoc test (**** = $p < 0.0001$).

B. Quantification of mScarlet-$A\beta_{1-42}$ fluorescence intensity of young adult (day 4 of life) and old adult (day 10 of life) nematodes of the $nA\beta_{1-42}$ strain. Confocal fluorescent images of three cohorts of total 33 - 38 nematodes were recorded, and fluorescence intensities were quantified by Fiji. Data are displayed as mean fluorescence intensity ± SD. Student's t-test with Welch's correction was performed to assess significance (**** = $p < 0.0001$).

C. Quantification of mScarlet-$A\beta_{1-42}$ fluorescence intensity of young adult (day 4 of life) and old adult (day 10 of life) nematodes of the $mA\beta_{1-42}$ strain. Confocal fluorescent images of three cohorts of total 38 - 40 nematodes were recorded, and fluorescence intensities were quantified by Fiji. Data are displayed as mean fluorescence intensity ± SD. Student's t-test with Welch's correction was performed to assess significance (ns = $p > 0.05$)

Disease Models & Mechanisms • Supplementary information

A

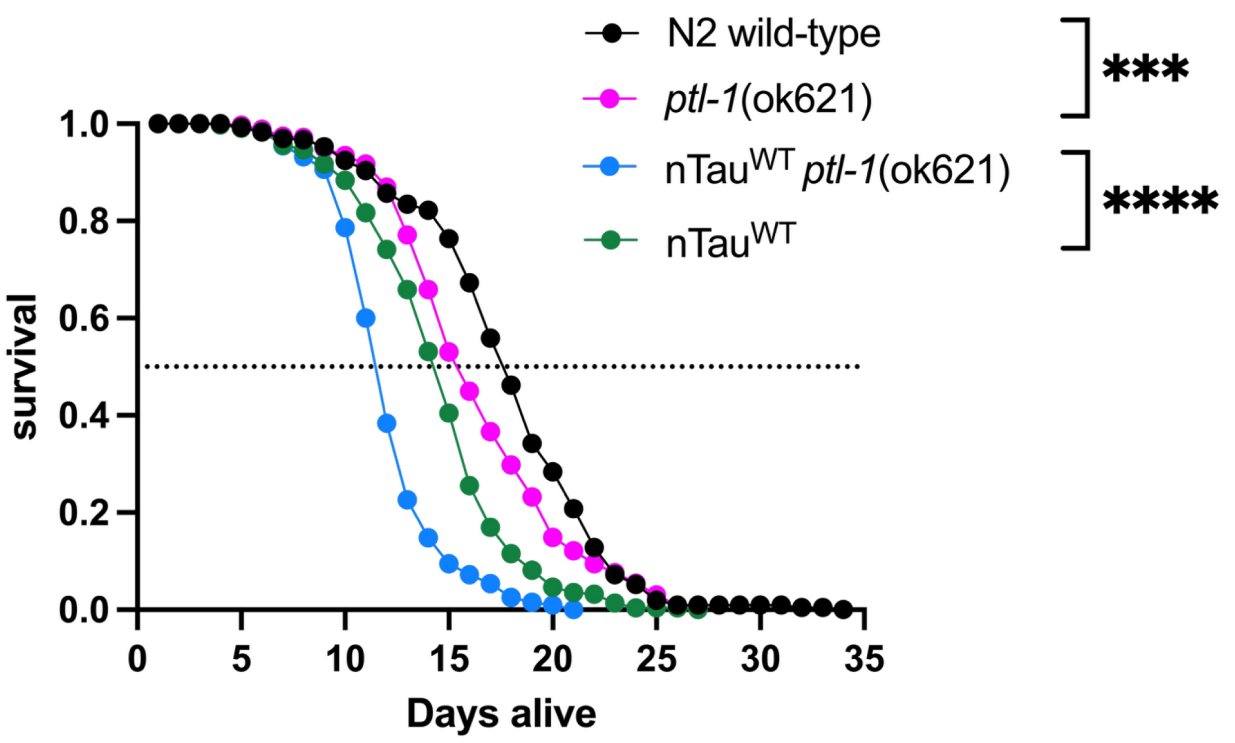

	ptl-1(ok621)	nTau^{WT}	N2 wild-type	ptl-1(ok621) nTau^{WT}
**Number of replicates**	3	3	3	3
**Total number of animals analysed**	336	323	297	324
**Total number of animals censored**	24	127	133	36
**Mean lifespan**	15.4	14.2	17.7	11.5
**Maximum Lifespan**	26	26	33	20
**p-values**	0.0001			
		< 1.0 * 10-10.		

B

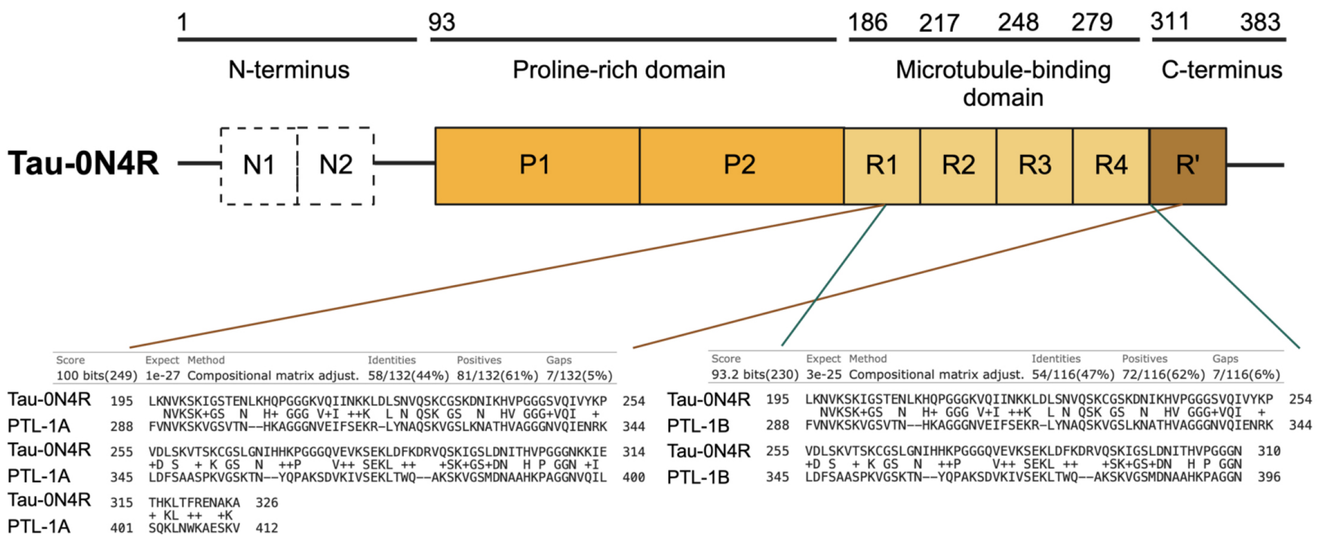

Fig. S8. Lifespan assay of *ptl-1* knock-out mutant and nTau^{WT} cross.

A. Assessment of the lifespan of N2 wild-type, *ptl-1*(*ok621*), nTau^{WT} x *ptl-1*(ok621) and nTau^{WT} animals. Graph shows the cumulative survival probability (survival) versus age (days alive) of N2 wild-type (black), *ptl-1*(*ok621*) (magenta), nTau^{WT} x *ptl-1*(*ok621*) (blue) and nTau^{WT} (green). Three independent cohorts of 100 - 150 nematodes each were analyzed, and significance was tested by log-rank test using Oasis2 online tool (**** = p ≤ 0.0001). The table below summarizes all parameter.

B. Protein sequences of human Tau-0N4R and both PTL-1 protein isoforms, PTL- 1A (left) and PTL-1B (right) respectively, align only within the MTBD (residues 186 to 311 of Tau-0N4R) with identities of 44 % and 47 %. NCBI reference sequences used for Protein BLAST search: NP_058518.1 (Tau 0N4R), AAB97090.1(PTL-1A) and AAC47829.1 (PTL-1B). Dashes (middle lane) denote gaps introduced into the sequences to maximize the alignment. Plus (middle lane) indicates residues that are similar but not identical.

Disease Models & Mechanisms • Supplementary information

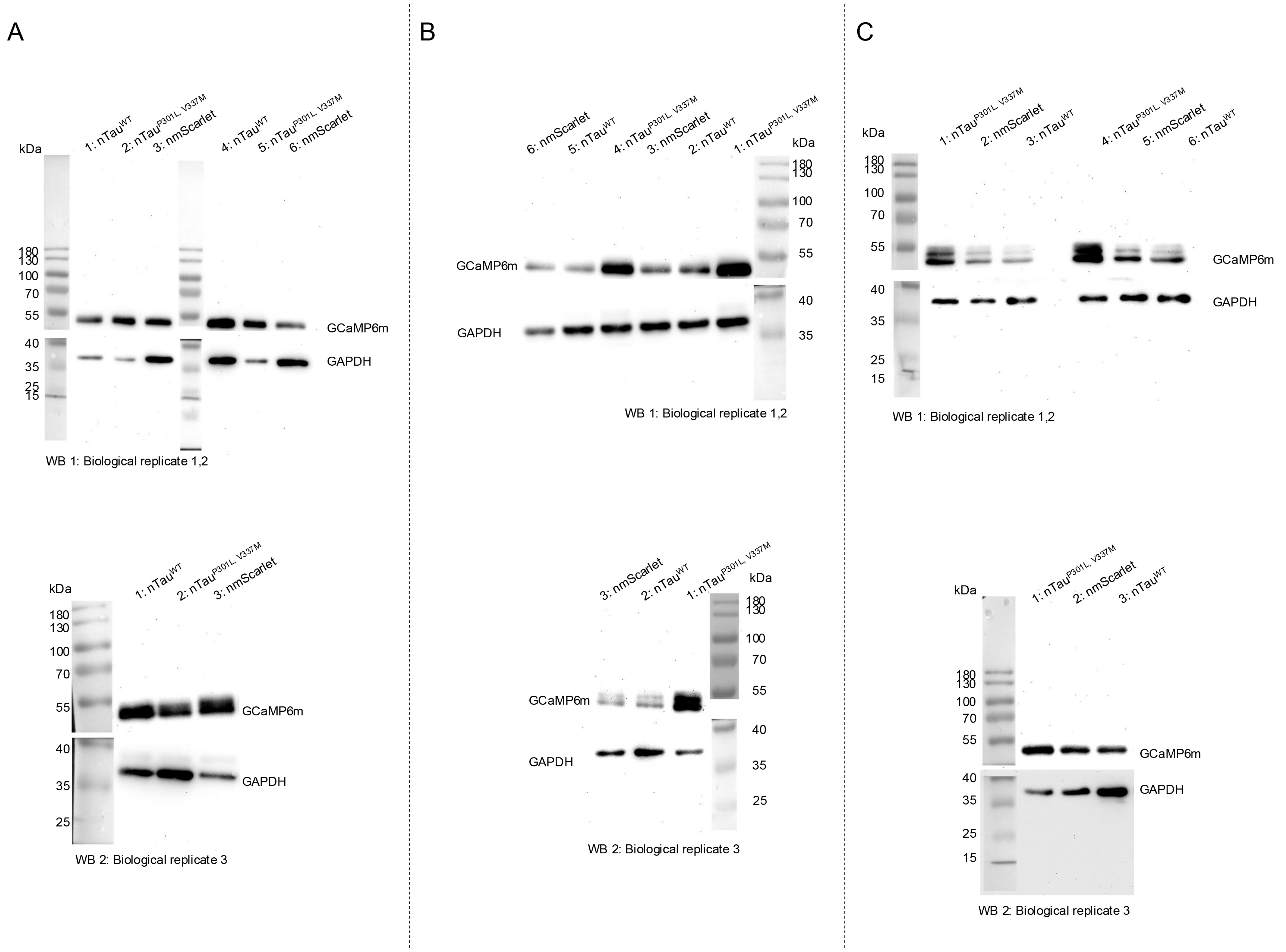

Fig. S9. Uncropped Western blots for the quantification of GCaMP6m protein levels in *C. elegans* expressing Tau and nmScarlet control strain. A. Western blots of the first, second and third biological replicate of crude protein lysates of young adult (day 4 of life) nTau^{WT} (lanes 1, 4), nTau^{P301L,V337M} (lanes 2, 5), and nmScarlet (lanes 3, 6) animals. Protein bands of GCaMP6m and GAPDH and molecular weights of protein ladder (kDa) are labeled.

B. Western blots of the first, second and third biological replicate of crude protein lysates of adult (day 7 of life) nTau^{WT} (lane 2, 5), nTau^{P301L,V337M} (lane 1, 4), and nmScarlet (lane 3, 6) animals. Protein bands of GCaMP6m and GAPDH and molecular weights of protein ladder (kDa) are labeled.

C. Western blots of the first, second and third biological replicate of crude protein lysates of old adult (day 10 of life) nTau^{WT} (lane 3, 6), nTau$^{P301L, V337M}$ (lane 1, 4), and nmScarlet (lanes 2, 4) animals. Protein bands of GCaMP6m and GAPDH and molecular weights of protein ladder (kDa) are labeled.

A

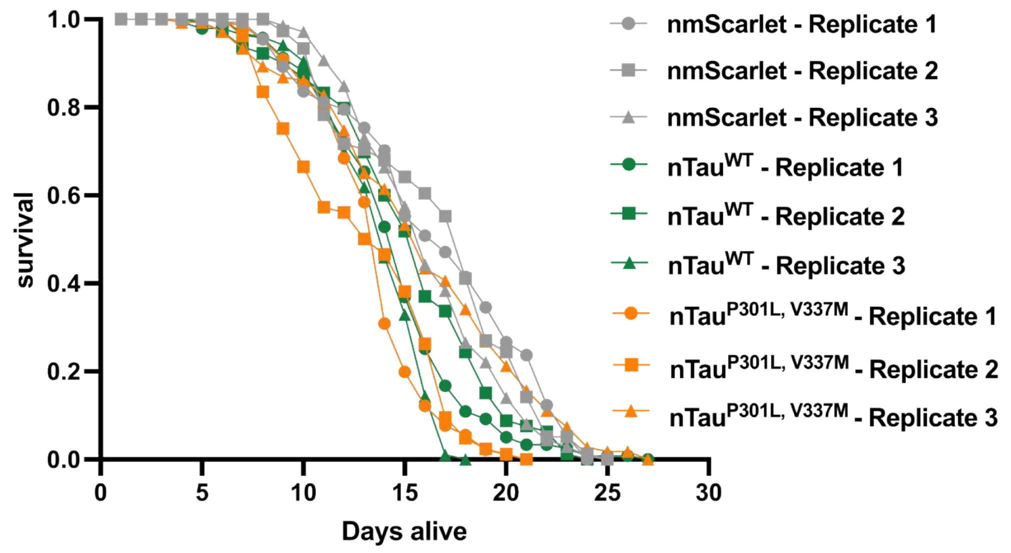

Days alive	survival nmScarlet Replicate 1	nmScarlet Replicate 2	nmScarlet Replicate 3	nTau$^{P301L, V337M}$ Replicate 1	nTau$^{P301L, V337M}$ Replicate 2	nTau$^{P301L, V337M}$ Replicate 3	nTau^{WT} Replicate 1	nTau^{WT} Replicate 2	nTau^{WT} Replicate 3
1	1.00	1.00	1.00	1.00	1.00	1.00	1.00	1.00	1.00
2	1.00	1.00	1.00	1.00	1.00	1.00	1.00	1.00	1.00
3	1.00	1.00	1.00	1.00	1.00	1.00	1.00	1.00	1.00
4	1.00	1.00	1.00	1.00	1.00	0.99	0.99	1.00	1.00
5	1.00	1.00	1.00	1.00	1.00	0.99	0.98	0.99	1.00
6	0.99	1.00	1.00	0.99	1.00	0.97	0.98	0.97	1.00
7	0.99	1.00	1.00	0.99	0.96	0.93	0.96	0.94	0.97
8	0.95	1.00	1.00	0.95	0.84	0.89	0.96	0.92	0.96
9	0.89	0.97	0.99	0.91	0.75	0.87	0.91	0.90	0.94
10	0.84	0.93	0.97	0.85	0.66	0.86	0.88	0.87	0.90
11	0.81	0.78	0.91	0.80	0.57	0.83	0.80	0.83	0.82
12	0.80	0.72	0.85	0.68	0.56	0.75	0.72	0.80	0.70
13	0.75	0.70	0.73	0.58	0.50	0.65	0.65	0.70	0.62
14	0.70	0.68	0.66	0.31	0.47	0.62	0.53	0.60	0.46
15	0.55	0.64	0.58	0.20	0.38	0.53	0.37	0.52	0.33
16	0.51	0.60	0.44	0.12	0.26	0.43	0.25	0.37	0.14
17	0.47	0.55	0.38	0.08	0.10	0.41	0.17	0.34	0.01
18	0.41	0.41	0.27	0.06	0.05	0.34	0.11	0.24	0.00
19	0.35	0.27	0.22	0.02	0.02	0.27	0.09	0.15	
20	0.27	0.24	0.14	0.01	0.01	0.21	0.05	0.09	
21	0.24	0.14	0.08	0.00	0.00	0.16	0.03	0.08	
22	0.12	0.05	0.04			0.11	0.03	0.06	
23	0.05	0.05	0.03			0.07	0.03	0.01	
24	0.00	0.01	0.01			0.03	0.01	0.00	
25		0.00	0.00			0.02	0.01		
26						0.02	0.01		
27						0.00	0.00		

	nTau^{P301L,V337M}			nTau^{WT}			nmScarlet		
No. Replicate	1	2	3	1	2	3	1	2	3
Total number of animals analysed	97	91	113	122	100	101	110	86	136
Total number of animals censored	53	59	37	28	50	49	40	94	14
Mean lifespan	13.4	13.8	15.4	14.2	15.3	13.8	16.5	17.6	15.6
Maximum Lifespan	20	20	26	26	23	17	23	24	24

B

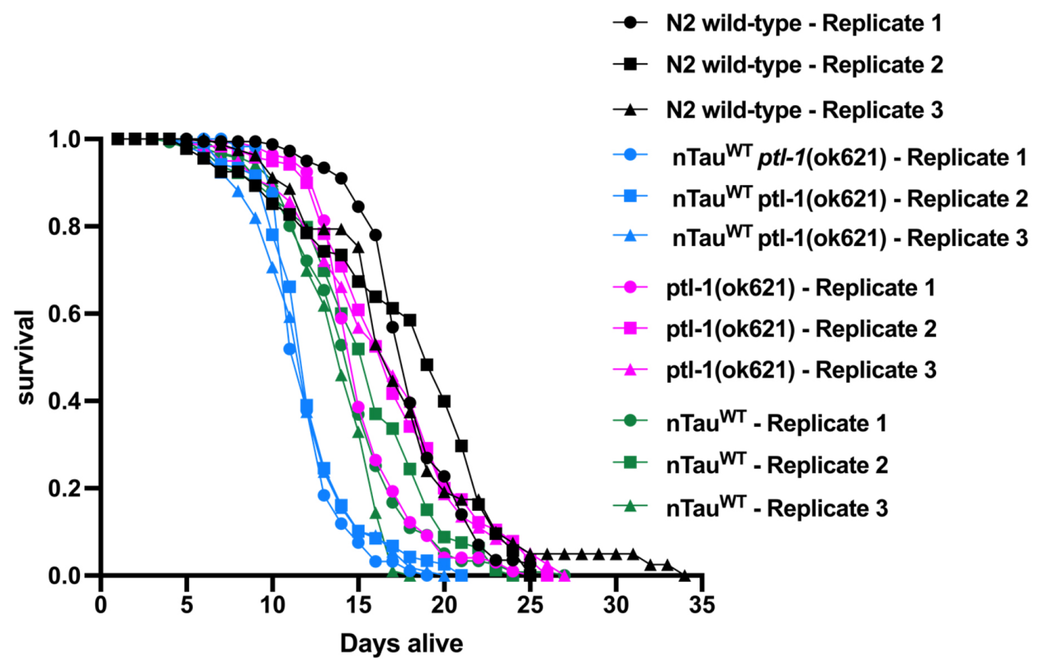

Days alive	survival N2 wild-type Replicate 1	N2 wild-type Replicate 2	N2 wild-type Replicate 3	nTau^{WT}ptl-1(ok621) Replicate 1	nTau^{WT}ptl-1(ok621) Replicate 2	nTau^{WT}ptl-1(ok621) Replicate 3	ptl-1(ok621) Replicate 1	ptl-1(ok621) Replicate 2	ptl-1(ok621) Replicate 3	nTau^{WT} Replicate 1	nTau^{WT} Replicate 2	nTau^{WT} Replicate 3
1	1.00	1.00	1.00	1.00	1	1	1.00	1	1	1.00	1.00	1.00
2	1.00	1.00	1.00	1.00	1	1	1.00	1	1	1.00	1.00	1.00
3	1.00	1.00	1.00	1.00	1	1	1.00	1	1	1.00	1.00	1.00
4	1.00	1.00	1.00	1.00	1	1	1.00	1	1	0.99	1.00	1.00
5	1.00	0.98	1.00	1.00	0.99	0.98	1.00	0.99	1	0.98	0.99	1.00
6	0.99	0.96	1.00	1.00	0.99	0.97	1.00	0.98	0.98	0.98	0.97	1.00
7	0.99	0.93	0.99	1.00	0.94	0.92	0.99	0.98	0.95	0.96	0.94	0.97
8	0.99	0.93	0.98	0.98	0.93	0.88	0.99	0.98	0.95	0.96	0.92	0.96
9	0.99	0.89	0.96	0.98	0.92	0.82	0.97	0.96	0.91	0.91	0.90	0.94
10	0.99	0.85	0.91	0.88	0.78	0.71	0.95	0.95	0.89	0.88	0.87	0.90
11	0.97	0.83	0.89	0.52	0.66	0.59	0.94	0.94	0.86	0.80	0.83	0.82
12	0.95	0.78	0.79	0.38	0.39	0.38	0.89	0.90	0.79	0.72	0.80	0.70
13	0.93	0.74	0.79	0.18	0.25	0.24	0.78	0.78	0.72	0.65	0.70	0.62
14	0.91	0.73	0.79	0.12	0.16	0.16	0.57	0.71	0.66	0.53	0.60	0.46
15	0.84	0.67	0.75	0.08	0.10	0.10	0.37	0.61	0.57	0.37	0.52	0.33
16	0.78	0.64	0.53	0.03	0.08	0.09	0.25	0.53	0.53	0.25	0.37	0.14
17	0.57	0.61	0.45	0.03	0.07	0.05	0.19	0.42	0.46	0.17	0.34	0.01
18	0.40	0.59	0.37	0.01	0.04	0.02	0.12	0.34	0.40	0.11	0.24	0.00
19	0.27	0.48	0.24	0.00	0.03	0.01	0.09	0.29	0.29	0.09	0.15	
20	0.23	0.40	0.19		0.03	0.00	0.04	0.20	0.19	0.05	0.09	
21	0.14	0.30	0.17		0.00		0.04	0.17	0.14	0.03	0.08	
22	0.07	0.16	0.17				0.04	0.12	0.11	0.03	0.06	
23	0.03	0.10	0.10				0.03	0.10	0.08	0.03	0.01	
24	0.03	0.06	0.07				0.01	0.08	0.07	0.01	0.00	
25	0.03	0.00	0.05				0.00	0.03	0.05	0.01		
26			0.05						0.03	0.01		
27			0.05						0.00	0.00		
28			0.05									
29			0.05									
30			0.05									
31			0.05									
32			0.02									
33			0.02									
34			0.00									

	ptl-1(ok621)			nTau^{WT}			N2 wild-type			nTau^{WT} ptl-1(ok621)		
No. Replicate	1	2	3	1	2	3	1	2	3	1	2	3
Total number of animals analysed	99	119	118	122	100	101	119	112	66	93	118	113
Total number of animals censored	21	1	2	28	50	49	61	38	34	27	2	7
Mean lifespan	14.5	16.2	16.4	14.2	15.3	13.8	17.4	18.9	16.5	11.2	11.6	11.4
Maximum lifespan	24	25	26	26	23	17	25	24	33	18	20	19

Fig. S10. Lifespan data as graph and tabulated form.

A. Lifespan data of 3 replicates each of nmScarlet (grey), nTau^{WT} (green) and nTau^{P301L,V337M} (orange) animals as graph and tables.

B. Lifespan data of 3 replicates each of N2 (wildtype), nTauWT, *ptl-1* (ok621) and nTauWT x *ptl-1* (ok621) as graph and tables.

Disease Models & Mechanisms • Supplementary information

Table S1. *C. elegans* strains and crossings used in this study

Strain list

Name	Systematic Name	Genotype	Source
m$A\beta_{1-42}$	JKM7	*jkIs7* [*myo-3p::Signalpeptide-Abeta(1-42)::hsp-3(IRES)::wrmScarlet-Abeta(1-42)::unc-54(3'UTR) + rps-0p::HygroR*]	Gallrein et al. 2021
mmScarlet	JKM9	*jkIs9* [*myo-3p::wrmScarlet::unc-54(3'UTR) + rps-0p::HygroR*]	Gallrein et al. 2021
nmScarlet	JKM12	*jkIs12* [*rgef-1p::hsp-3(IRES)::wrmScarlet*]	Pigazzini et al. 2021
unc-13	MT8004	*unc-13* (*n2813*) I.	CGC
wild type	N2	C. elegans wild isolate	CGC
n$A\beta_{1-42}$	JKM2	*jkIs2* [*rgef-1p::Signalpeptide-Abeta(1-42)::hsp-3(IRES)::wrmScarlet-Abeta(1-42)::unc-54(3'UTR) + rps-0p::HygroR*]	Gallrein et al. 2021
nGCaMP6m	JKM280	*jkIs16* [*rgef-1p::GCaMP6m*]	this study
$nTau^{P301L,\ V337M}$	JKM41	*jkIs14* [*rgef-1p::TauFL0N4R(P301L, V337M)::hsp-3(IRES)::wrmScarlet-TauFL0N4R(P301L, V337M)::unc-54(3'UTR)+rps-0p::HygroR*]	this study
$nTau^{WT}$	JKM40	*jkIs13* [*rgef-1p::TauFL0N4R::hsp-3(IRES)::wrmScarlet-TauFL0N4R::unc-54(3'UTR)+rps-0p::HygroR*]	this study
ptl-1	RB809	*ptl-1*(*ok621*) III.	CGC
coelomocyte marker	ZIM1048	*mzmIs4* [*unc-31p::NLSGCaMP5Kf +unc-122p::GFP*]; *lite-1* (*ce314*) X.	Nichols et al., 2017
mec-4::GFP	SK4005	*zdIs5* [*mec-4::GFP + lin-15(+)*]	Clark and Chiu (2003)

Crossings

Name	Systematic Name	*Genotype*	Source
m$A\beta_{1-42}$×nGCaMP6m	JKM281	*jkIs7; jkIs16*	this study
mmScarlet×nGCaMP6m	JKM282	*jkIs9; jkIs16*	this study
n$A\beta_{1-42}$×nGCaMP6m	JKM152	*jkIs2; jskIs16*	this study
nGCaMP6m×MT8004	JKM155	*jkIs16; unc-11(n2813)*	this study
nmScarlet×nGCaMP6m	JKM20	*jkIs12; jkIs16*	this study
$nTau^{P301L,\ V337M}$×nGCaMP6m	JKM151	*jkIs14; jkIs16*	this study
$nTau^{P301L,\ V337M}$×ZIM1048	JKM154	*jkIs14; mzmIs4*	this study
$nTau^{WT}$×nGCaMP6m	JKM150	*jkIs13; jkIs16*	this study
$nTau^{WT}$×RB809	JKM283	*jkIs13; ptl-1(ok621)*	this study
$nTau^{WT}$×ZIM1048	JKM153	*jkIs13; mzmIs4*	this study
nmScarlet×mec-4::GFP	JKM188	*jkIs12; zdIs5*	this study
$nTau^{WT}$ ×mec-4::GFP	JKM220	*jkIs13; zdIs5*	this study
$nTau^{P301L,\ V337M}$ ×mec-4::GFP	JKM187	*jkIs14; zdIs5*	this study

CGC: Caenorhabditis Genetics Center, University of Minnesota, USA (https://cgc.umn.edu).

Disease Models & Mechanisms • Supplementary information
